# Specific inhibition of fibroblast growth factor receptor 1 signaling by a DNA aptamer

**DOI:** 10.1016/j.omtn.2024.102405

**Published:** 2024-11-28

**Authors:** Vladimira Zlinska, Zuzana Feketova, Aleksandra Czyrek, Julia Chudzian, Martina Lenarcic Zivkovic, Vlad-Constantin Ursachi, Pooja Dudeja, Bohumil Fafilek, Jan Rynes, Gustavo Rico-Llanos, Adolf Koudelka, Tanaya Roy, Martyna Biadun, Vendula Raskova, Katerina Svozilova, Michaela Stroblova, Mateusz Krzyscik, Kalina Hristova, Daniel Krowarsch, Silvie Foldynova-Trantirkova, Malgorzata Zakrzewska, Lukas Trantirek, Pavel Krejci

**Affiliations:** 1 Central European Institute of Technology, Masaryk University, 625 00 Brno, Czechia; 2 National Centre for Biomolecular Research, Masaryk University, 625 00 Brno, Czechia; 3 Department of Biology, Faculty of Medicine, Masaryk University, 62500 Brno, Czechia; 4 International Clinical Research Center, St. Anne’s University Hospital, 65691 Brno, Czechia; 5 Department of Protein Engineering, University of Wroclaw, 50-383 Wroclaw, Poland; 6 Slovenian NMR Centre, National Institute of Chemistry, 1000 Ljubljana, Slovenia; 7 Institute of Animal Physiology and Genetics of the CAS, 60200 Brno, Czechia; 8 Department of Materials Science and Engineering, Institute for NanoBioTechnology and Program in Molecular Biophysics, Johns Hopkins University, Baltimore, MD 21218, USA; 9 Department of Protein Biotechnology, University of Wroclaw, 50-383 Wroclaw, Poland

**Keywords:** MT: Oligonucleotides: Therapies and Applications, FGFR signaling, FGFR1, inhibitor, DNA aptamer, extracellular domain, skeletal dysplasia, craniosynostosis

## Abstract

Impaired fibroblast growth factor receptor (FGFR) signaling is associated with many human conditions, including growth disorders, degenerative diseases, and cancer. Current FGFR therapeutics are based on chemical inhibitors of FGFR tyrosine kinase activity (TKIs). However, FGFR TKIs are limited in their target specificity as they generally inhibit all FGFRs and other receptor tyrosine kinases. In the search for specific inhibitors of human FGFR1, we identified VZ23, a DNA aptamer that binds to FGFR1b and FGFR1c with a K_D_ of 55 nM and 162 nM, respectively, but not to the other FGFR variants (FGFR2b, FGFR2c, FGFR3b, FGFR3c, FGFR4). In cells, VZ23 inhibited the activation of downstream FGFR1 signaling and FGFR1-mediated regulation of cellular senescence, proliferation, and extracellular matrix homeostasis. Consistent with the specificity toward FGFR1 observed *in vitro*, VZ23 did not inhibit FGFR2-4 signaling in cells. We show that the VZ23 inhibits FGFR1 signaling in the presence of cognate fibroblast growth factor (FGF) ligands and its inhibitory activity is linked to its capacity to form unusual G-quadruplex structure. Our data suggest that targeting FGFR1 with DNA aptamers could be an effective alternative to TKIs for treating impaired FGFR1 signaling in human craniosynostoses.

## Graphical abstract

**Figure:**
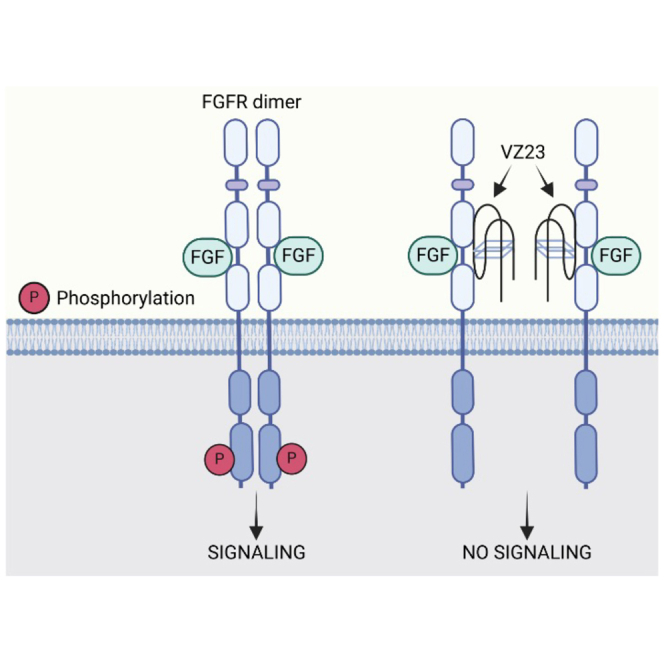

## Teaser

Krejci and colleagues developed a DNA-based aptamer that specifically inhibits the activation of downstream FGFR1 signaling and FGFR1-mediated regulation of cellular senescence, proliferation, and extracellular matrix homeostasis in the presence of FGF ligands. Their data suggest that targeting FGFR1 with aptamers could be a promising strategy for treating human craniosynostoses.

## Introduction

The fibroblast growth factor (FGF) family consists of 18 proteins that act as morphogens, growth factors, and metabolic hormones to regulate critical physiological processes during development and life.^1^ FGFs signal by binding and activating FGF-receptor tyrosine kinases (FGFRs). In mammals, there are four FGFRs (FGFR1-4) that further diversify into the "b" or "c" variants by alternative splicing in the extracellular immunoglobulin (Ig)-like domain 3, increasing the number of functionally active FGFR variants to seven (FGFR1b, FGFR1c, FGFR2b, FGFR2c, FGFR3b, FGFR3c, FGFR4).^2^ Since the Ig3 domain is involved in ligand binding, the seven FGFR variants show differences in the selection of their cognate FGFs. Experimental studies confirm 62 of the 126 theoretically possible FGF-FGFR interactions between the seven FGFRs and 18 FGF ligands.^3^^,^^4^

In mammals, FGF-FGFR interactions control most developmental processes, including blastocyst formation, gastrulation, and morphogenesis of lungs, limbs, and brain.^5^^,^^6^ Impaired FGFR signaling is associated with many pathological conditions, including growth disorders, degenerative diseases, and cancer.^7^ Current therapeutic approaches for targeting FGFR signaling are based on small molecule inhibitors of FGFR catalytic activity (tyrosine kinase inhibitors [TKIs]). Fifteen TKIs are currently in clinical trials for tumors caused by lesions in FGFR genes, such as FGFR amplifications, activating mutations, and fusion oncogenes involving FGFRs (Table S1). TKIs inhibit FGFR activation by preventing ATP binding to the kinase domain, except for futibatinib and RLY4008, which form a covalent bond with the cysteine residue in the phosphate binding loop of the FGFR kinase domain.^8^^,^^9^

Most FGFR TKIs exhibit low specificity among the FGFR1-4 variants, inhibiting most of the 62 FGF-FGFR interactions *in vivo*. In addition, FGFR TKIs also inhibit non-FGFR receptor tyrosine kinases, such as vascular endothelial growth factor receptor, platelet-derived growth factor receptor, discoidin domain receptor, and hepatocyte growth factor receptor (Table S1). The low selectivity among FGFRs and off-target activity contribute to the side effects and toxicity of FGFR-TKI therapies, thus limiting their use.

Recently, aptamer technology has yielded more specific inhibitors of FGFR signaling. For example, iR3, an RNA aptamer that specifically inhibits FGFR3^10^; Apt_46, a DNA aptamer that inhibits FGFR2b but not the other FGFR variants^11^; or RBM-007, an RNA aptamer that neutralizes the FGF ligand FGF2. RBM-007 is currently in clinical trials for achondroplasia and exudative age-related macular degeneration, demonstrating the therapeutic potential of aptamers for the targeted inhibition of FGFR signaling.^12^^,^^13^

## Results

In the search for a specific inhibitor of FGFR1 signaling, the extracellular domain (Arg22-Glu285) of human FGFR1c fused to the Fc domain of IgG1 was immobilized on protein G magnetic beads and used as bait to screen a commercial library of 10^15^ 76-nucleotide (nt) DNA oligonucleotides containing 40-nt long randomized region. A total of six rounds of SELEX were performed (Table S2), including negative selection against empty protein G in each round and against the extracellular domain of the cMET receptor tyrosine kinase in the final two rounds. A total of 10 aptamers found in the sixth SELEX round (labeled A–J) (Table S3) were cloned, sequenced, and tested for biological activity.

Rat chondrosarcoma (RCS) cells represent an established model for FGF signaling used to describe the mechanisms of FGF-mediated regulation of cell proliferation, differentiation, senescence, and others.^14^^,^^15^^,^^16^ Since wild-type RCS cells express four FGFRs (FGFR1c, FGFR2c, FGFR3c, FGFR4), we used CRISPR-Cas9 to delete *Fgfr2-4*, resulting in RCS cells expressing only FGFR1c (RCS-FGFR1c) (Figures S1A and S1B). RCS-FGFR1c cells were treated with the cognate FGFR1c ligand FGF1, and activation of the FGFR1c downstream signaling, RAS-ERK-MAP kinase (extracellular signal-regulated kinase [ERK] pathway), was determined. Aptamers A–C, E–H, and J had no effect on FGF1-mediated induction of ERK activation, whereas aptamers D and I showed partial (D) or complete (I) inhibition of ERK activity (Figure S2). Aptamer I was designated VZ23 and further characterized.

**Figure 1:**
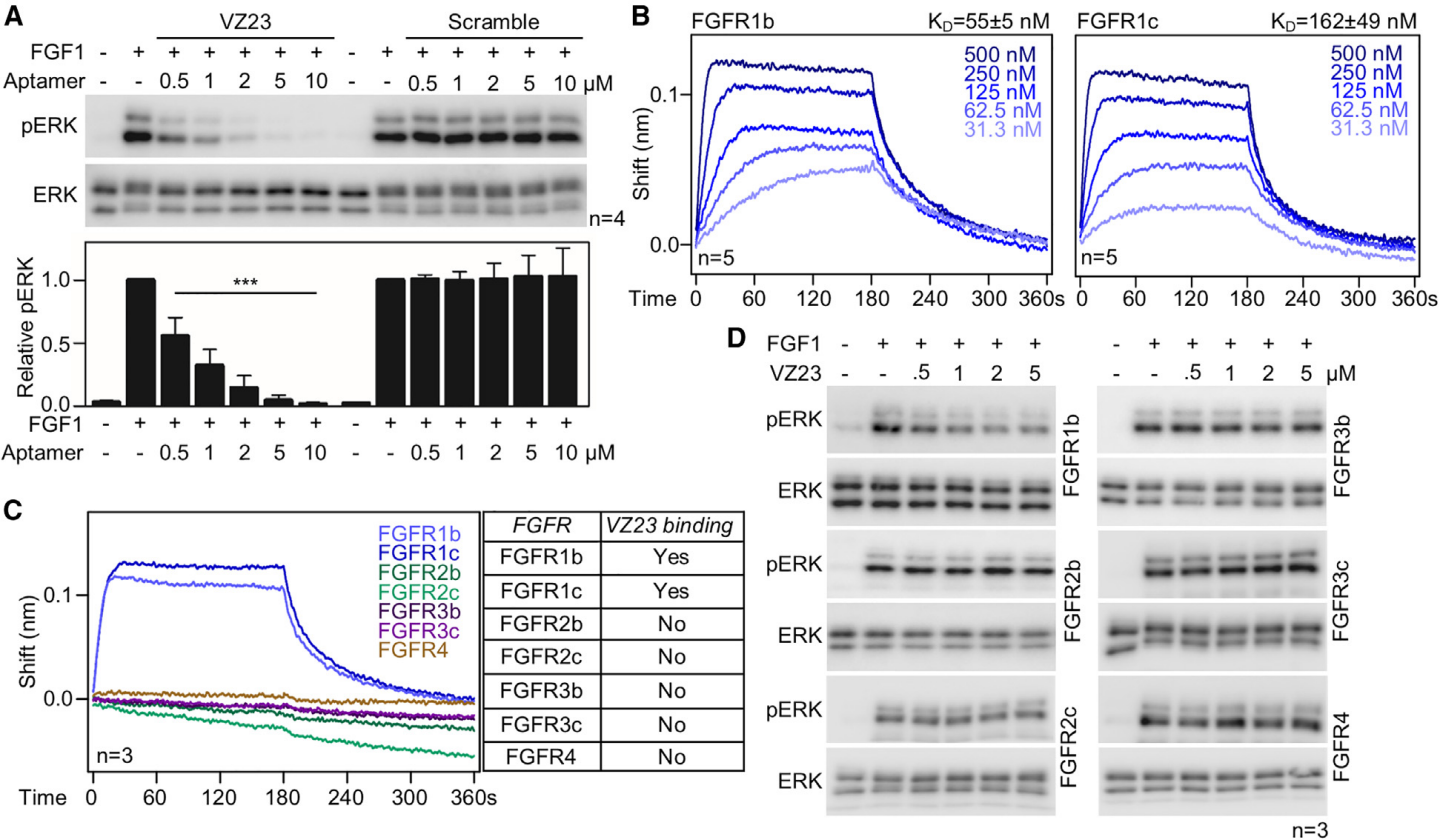
Functional characterization of VZ23 (A) RCS-FGFR1c (rat) cells were treated with VZ23 30 min prior to addition of FGF1 (5 ng/mL) for 1 h and analyzed for phosphorylated (p) ERK by western blot; total ERK served as loading control. pERK signal was quantified and plotted (mean ± SD; ANOVA, ∗∗∗*p* < 0.001; n, number of independent experiments). VZ23 inhibits FGF1-mediated activation of the ERK pathway; scramble aptamer has no effect. (B and C) BLI analysis of VZ23 binding on human FGFRs. VZ23 binds to FGFR1b and FGFR1c, but not to the other FGFR variants (table). (D) VZ23 inhibits FGF1-mediated activation of ERK in RCS-FGFR1b cells but not in RCS cells expressing rat (FGFR2c, FGFR3c, FGFR4) or human (FGFR1b, FGFR2b, FGFR3b) FGFR variants.

Initial experiments investigating the effect of VZ23 on FGF1-mediated activation of the ERK pathway were performed with the 40-nt long VZ23 sequence plus two 18-nt flanking primer sequences used for PCR amplification during SELEX (Table S3). Strong inhibition of FGF1-mediated ERK activation was observed for VZ23 without primer sequences (Figure S3A); no activity was found for scramble or reverse DNA sequences of VZ23 (Figures 1A and S3B). No inhibitory activity of VZ23 on the signaling of the insulin receptor, stem cell growth factor receptor, hepatocyte growth factor receptor and epidermal growth factor receptor was observed (Figure S4).

Next, we investigated the interactions of VZ23 with FGFRs using biolayer interferometry (BLI). Recombinant human full-length extracellular domains of FGFR1b, FGFR1c, FGFR2b, FGFR2c, FGFR3b, FGFR3c and FGFR4 fused to the Fc domain of IgG1 were prepared as previously described^17^^,^^18^ and immobilized on ProtA sensors. Kinetic measurements followed by global fitting with the 1:1 ligand model and steady-state analysis showed that VZ23 associates with FGFR1 variants with K_D_ values of 54.6 ± 5.4 nM (*n* = 5) for FGFR1b and 162 ± 48.7 nM (*n* = 5) for FGFR1c (Figures 1B and S3C). No association of VZ23 with FGFR2b, FGFR2c, FGFR3b, FGFR3c, or FGFR4 was detected (Figure 1C).

To examine the specificity of VZ23 toward FGFRs in cells, RCS cells expressing only FGFR2c (RCS-FGFR2c), FGFR3c (RCS-FGFR3c), and FGFR4 (RCS-FGFR4) were generated from wild-type RCS cells by CRISPR-Cas9 deletion of the other endogenous FGFRs (Figures S1A and S1B). The RCS cells expressing single human FGFR variants were derived from wild-type RCS cells in which the endogenous *Fgfr1-4* genes were inactivated by CRISPR-Cas9 (RCS-FGFR1-4 *null*). These cells were stably transfected with vectors containing full-length human *FGFR1b*, *FGFR1c*, *FGFR2b*, *FGFR2c*, *FGFR3b*, *FGFR3c*, or *FGFR4* using PiggyBac transposase (Figure S1C). VZ23 inhibited FGF1-mediated ERK activation in RCS-FGFR1b cells; however, this inhibition was weaker than FGFR1c (Figures 1D and S3D). Of note, VZ23 did not inhibit FGF1-mediated ERK activation in cells expressing FGFR2b, FGFR2c, FGFR3b, FGFR3c, and FGFR4.

**Figure 2:**
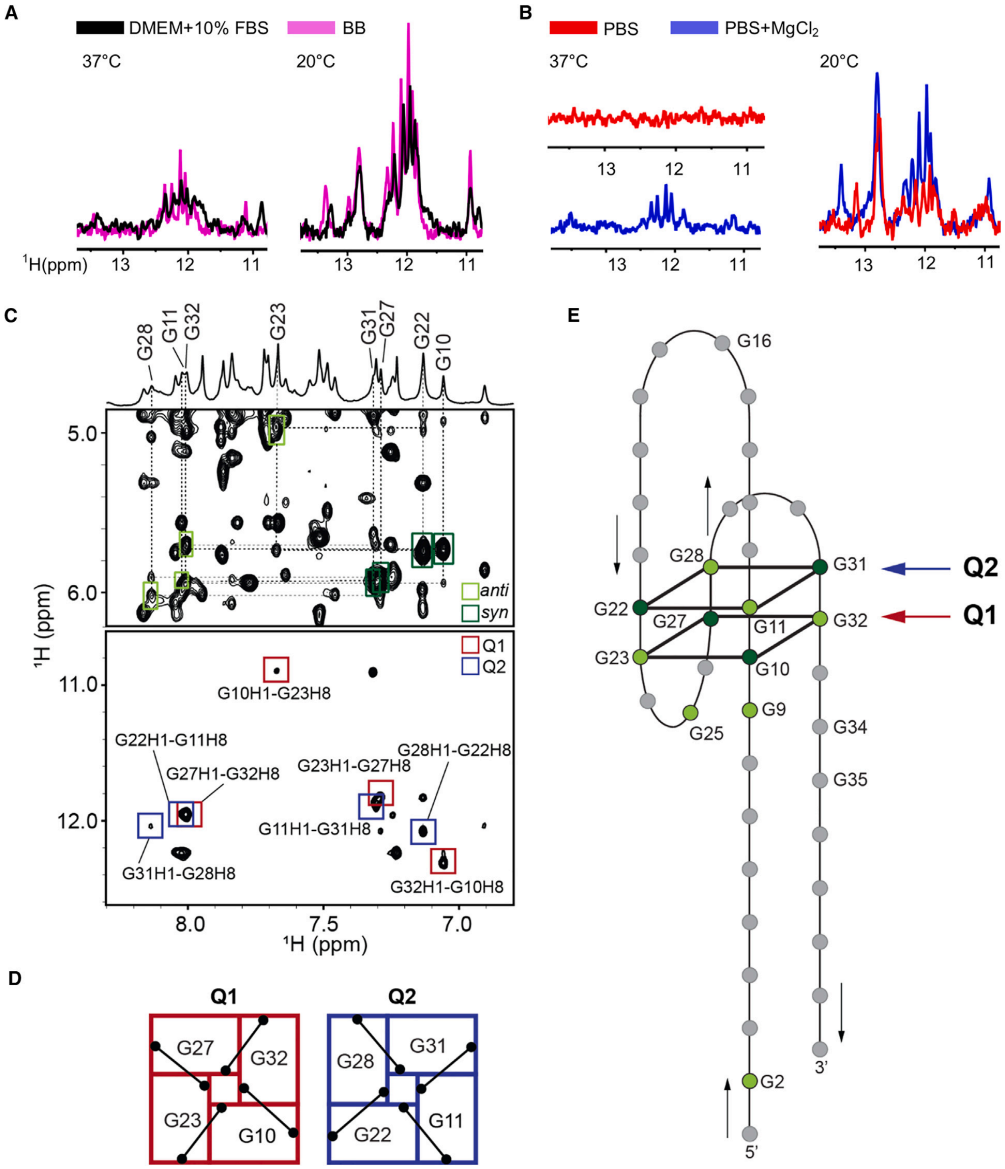
Structural characterization of VZ23 (A and B) Imino regions of 1D ^1^H NMR spectra of VZ23 acquired in DMEM supplemented with 10% FBS (black), binding buffer (pink), PBS (red), and PBS supplemented with 2 mM MgCl_2_ (blue) at 37°C and 20°C. The spectra suggest that VZ23 adopts a non-canonical DNA structure stabilized by Watson-Crick and Hoogsteen base pairs and depends on the structure stability on Mg^2+^. (C) Anomeric-aromatic and imino-aromatic regions of ^1^H–^1^H 2D NOESY NMR spectrum (mixing time 250 ms) of VZ23 were used to assess glycosidic conformations of guanines from G-quartets and intra-quartet H1-H8 NOE connectivities, respectively. (D) G-quartets 1 and 2 (Q1 and Q2) with the corresponding H1-H8 NOE connectivities observed on the ^1^H–^1^H 2D NOESY spectrum (black lines). (E) Schematic presentation of G-quadruplex-based topology adopted by VZ23 with antiparallel orientations of G-strands, long 5′- and 3′-tails, and three edgewise loops, respectively. Residues in G-quartets 1 and 2 (Q1 and Q2) adopting *anti*- and *syn*-glycosidic conformations are colored light and dark green, respectively. The antiparallel orientation of the G-strand is emphasized with black arrows.

Nuclear magnetic resonance (NMR) and circular dichroism (CD) were used to study the structure of VZ23. The imino region of the ^1^H 1D NMR spectra of VZ23 acquired at 20°C and 37°C in SELEX binding buffer and fetal bovine serum (FBS)-supplemented DMEM showed almost identical patterns characterized by multiple signals between 10 and 14 ppm (Figure 2A), suggesting that VZ23 adopts a non-canonical DNA structure stabilized by Watson-Crick and Hoogsteen base pairs. In contrast, the corresponding NMR spectrum of VZ23 acquired in the PBS buffer at 37°C showed no signals (Figure 2B), which indicated that the structure did not fold under these conditions. However, the pattern of the NMR spectrum of VZ23 in the PBS buffer supplemented with 2 mM Mg^2+^ closely resembled those observed in the binding buffer and FBS-supplemented DMEM (Figure 2B), suggesting that Mg^2+^ serves to stabilize the VZ23 structure (Figure S5A). This observation was further supported by the CD melting profiles of VZ23 obtained in PBS supplemented with 2 mM Mg^2+^, revealing a structure melting temperature approximately 10°C higher than that observed in PBS lacking Mg^2+^ (Figure S5B). The analysis of imino-aromatic connectivities in ^1^H–^1^H 2D nuclear Overhauser effect spectroscopy (NOESY) (Figures 2C and S6) revealed that VZ23 adopts antiparallel G-quadruplex-like folding topology (Figures 2D and 2E). Remarkably, stabilization of VZ23 by Mg^2+^ leads to a decrease in the K_D_ of binding of VZ23 to FGFR1, by 45% for FGFR1b (54.6 ± 5.4 vs. 29.8 ± 9 nM with Mg^2+^, *n* = 5) and 60% for FGFR1c (162 ± 48.7 vs. 65.4 ± 5.5 nM with Mg^2+^, *n* = 5) (Figures S5C and S5D).

**Figure 3:**
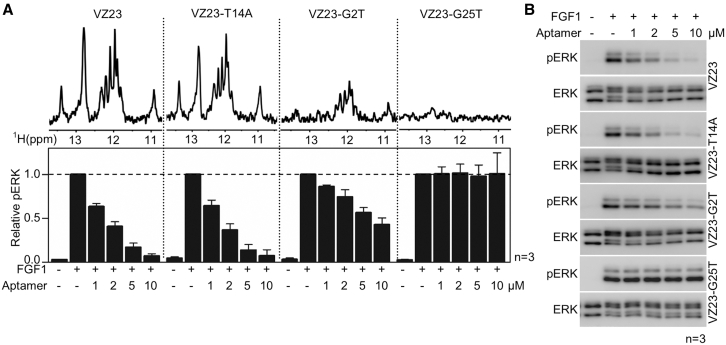
Effect of VZ23 stability on its inhibitory activity (A) Imino regions of 1D ^1^H NMR spectra of VZ23 mutants (T14A, G2T, and G25T). The decreased signal intensities in the mutants' NMR spectra, compared with the spectrum of wild-type VZ23, reflect the impaired capacity of the mutants to fold (upper graph). (B) VZ23-T14A, G2T, and VZ23-G25T inhibitory effect on FGF1-mediated activation of ERK signaling in RCS-FGFR1c (rat) cells. pERK signal was quantified and plotted (A, lower graph, mean ± SD; n, number of independent experiments). Collectively, the data in (A) and (B) show that the stability of the VZ23 structure positively correlates with aptamer inhibitory activity, i.e., the higher the aptamer’s stability, the higher its inhibitory effect.

To investigate the relationship between the structural stability of VZ23 and biological activity, single nucleotide substitutions G2T, T14A, and G25T were introduced into VZ23. Analysis of the NMR spectra of these VZ23 mutants revealed no effect of the T14A substitution on the VZ23 structure, a moderate destabilizing impact of G2T, and a significant destabilizing impact of the G25T substitution (Figure 3A). The relative stability of the VZ23 mutants correlated with their biological activity in RCS-FGFR1c cells. VZ23-G25T showed no inhibitory effect on FGF1-mediated activation of ERK signaling, G2T showed only partial inhibition compared with wild-type VZ23, while VZ23-T14A inhibited FGF1-mediated ERK activity to a similar extent as wild-type VZ23 (Figure 3B). Overall, these results highlight the importance of the secondary structure of VZ23 for its binding to FGFR1 and inhibition of FGFR1 signaling in cells.

**Figure 4:**
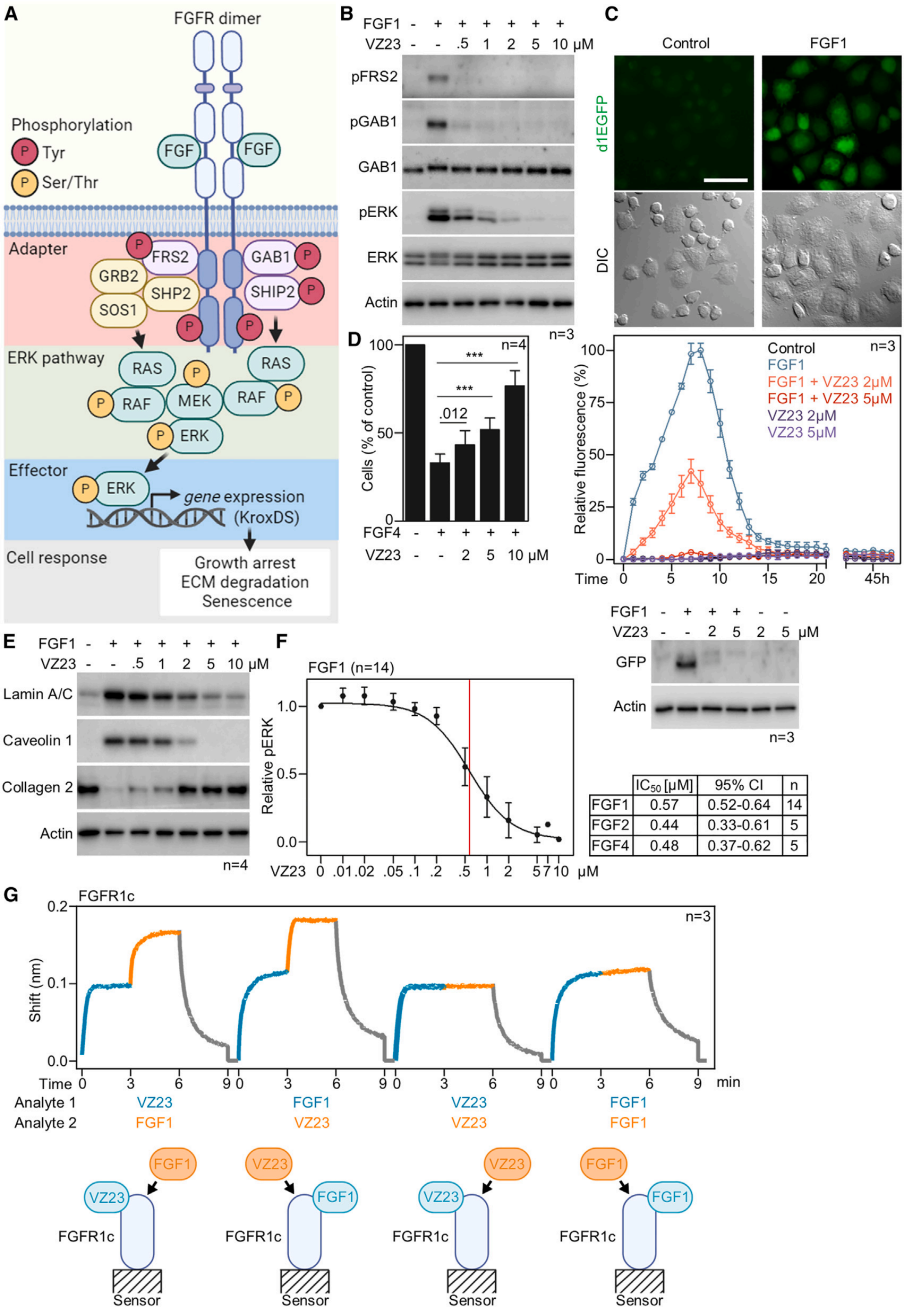
Inhibitory effect of VZ23 on FGFR1 signaling (A) FGF binding activates FGFR tyrosine kinase activity, leading to tyrosine phosphorylation of the adapter proteins FRS2, GAB1, and SHIP2. This creates docking sites for the GRB2:SHP2:SOS1 complexes, which transmit the signal to the RAS-RAF-MEK-ERK pathway; activated ERK induces gene transcription, leading to growth arrest, degradation of the extracellular matrix (ECM) and induction of premature senescence in RCS cells. (B) FGF1-mediated phosphorylation (p) of FRS2 and GAB1 in RCS-FGFR1c (rat) cells is inhibited by VZ23 (n, number of independent experiments). (C) FGF1-mediated induction of GFP expression in RCS-FGFR1c cells stably expressing the KroxDS reporter (top images; bar, 150 μm). VZ23 inhibits FGF1-mediated induction of GFP expression as observed by live cell imaging of GFP fluorescence over 45 h (middle graph). Western blot validation of VZ23 effect on GFP induction in cells treated with FGF1 for 4.5h, actin served as loading control (bottom panel). (D) Growth arrest in RCS-FGFR1c cells induced by 72 h of treatment with FGF4 was abrogated by VZ23 (mean ± SD; ANOVA, ∗∗∗*p* < 0.001). (E) Induction of premature senescence in cells treated with FGF1 for 24 h, as evidenced by upregulation of lamin A/C and caveolin 1 and degradation of ECM (loss of collagen 2). Senescence was reversed by VZ23. (F) Calculation of the IC_50_ for the effect of VZ23 on FGF1-mediated ERK activation in RCS-FGFR1c cells (graph, mean ± SD). Similar IC_50_ values were found for FGF2 and FGF4 (table; Figure S7A). (G) BLI experiments to evaluate the binding of FGF1 to the already formed VZ23:FGFR1c complex or the binding of VZ23 to the already formed FGF1:FGFR1c complex.

FGF binding promotes FGFR dimerization and ensures the optimal positioning of the cytosolic tyrosine kinase domains, allowing reciprocal phosphorylation within the FGFR dimer.^19^ Activated FGFRs phosphorylate the adapter proteins FRS2, GAB1, and SHIP2.^20^^,^^21^ This creates docking sites for the GRB2:SOS1 and SHP2:GRB2:SOS1 complexes, which bring SOS1 into the vicinity of the small GTPase RAS; SOS1 activates RAS by exchanging GDP for GTP.^22^ The signal is then relayed to the RAF-MEK-ERK signaling module via sequential Ser/Thr phosphorylation. Active ERK triggers changes in gene transcription that underlie the cellular response to FGF (Figure 4A).

To gain insight into the mechanism by which VZ23 inhibits FGFR1 signaling *in vivo*, we focused on the mechanisms of downstream FGFR1 signaling transduction in RCS-FGFR1c cells. In addition to ERK, the FGFR1c-mediated tyrosine phosphorylation of FRS2 and GAB1 adapters was also inhibited by VZ23 (Figure 4B). To assess the transcriptional activity of the FGFR1-ERK signaling, we developed an unstable variant of the pKrox24(MapERK)dsRed transcriptional reporter^23^ by replacing the dsRed with destabilized d1EGFP complemented by the 3′-UTR of the mouse *Egr1* gene (destabilized pKrox24(MapERK)d1EGFP; KroxDS). RCS-FGFR1c cells stably expressing KroxDS were treated with VZ23 and FGF1, and transactivation of KroxDS was monitored for up to 45 h by automated microscopy. FGF1 caused rapid KroxDS induction, which peaked at approximately 7–8 h and persisted for up to 14 h (Figure 4C); KroxDS transactivation was inhibited by VZ23.

RCS cells respond to FGF treatment with several cellular phenotypes, such as growth arrest, degradation of the extracellular matrix (ECM), and induction of premature senescence. Treatment of RCS-FGFR1c cells with FGF4 for 72 h resulted in a potent inhibition of cell proliferation, which was reversed by VZ23 (Figure 4D). FGF1-mediated induction of ECM degradation in RCS-FGFR1c cells was manifested by loss of collagen 2, whereas induction of senescence was shown by upregulation of senescence markers caveolin 1 and lamin A/C (Figure 4E)^15^; both phenotypes were blocked by VZ23.

In addition to FGF1, FGFR1c can also be activated by FGF2 and FGF4.^3^ Treatment of RCS-FGFR1c cells with FGF2 or FGF4 resulted in similar ERK activation as FGF1; inhibition by VZ23 was also similar for the three ligands, with IC_50_ values of 0.57, 0.44, and 0.48 μM for FGF1, FGF2, and FGF4, respectively (Figures 4F and S7A). These data suggest that VZ23 does not inhibit FGFR1c signaling in an FGF-dependent manner.

Competitive binding experiments show that VZ23 does not prevent the interaction of FGF1 with FGFR1b or FGFR1c (Figures 4G and S7B). Moreover, VZ23 can associate with already formed FGF1:FGFR1b and FGF1:FGFR1c complexes. The extracellular part of FGFR1 consists of three Ig-like domains that are involved in FGF binding (Ig2, Ig3) or have a regulatory function (Ig1)^24^^,^^25^ (Figure S8A). Deletion of Ig1 did not abolish the binding of VZ23 to FGFR1 (Figure S8B). Together, our data support the hypothesis that the VZ23 binding site on FGFR1 is distinct from the FGF binding site and does not involve Ig1.

Finally, we compared the effect of VZ23 with zoligratinib, an established FGFR TKI currently being investigated in clinical trials for the treatment of cancers caused by lesions in FGFRs (Table S1). Zoligratinib inhibited FGF-mediated ERK activation in RCS cells expressing only endogenous FGFR1c, FGFR2c, FGFR3c, and FGFR4 (Figure S9A). In similar experiments, VZ23 inhibited only FGFR1c signaling, demonstrating its major advantage over FGFR TKIs, namely its selectivity for a single FGFR variant, FGFR1 (Figure 1). We calculated the IC_50_ for zoligratinib-mediated inhibition of FGF1-FGFR1c signaling to be 0.13 μM (Figure S9B). The efficacy of zoligratinib appears to be higher than that of VZ23, which has an IC_50_ of 0.57 μM for FGF1-FGFR1c (Figure 4F). We find the efficacy of VZ23 encouraging, considering that it is a first-generation FGFR1 aptamer, unlike zoligratinib, which has already been optimized for its function. Ongoing research aims to understand the structure of VZ23 and produce more stable and active VZ23 derivatives.

## Discussion

Using SELEX, we identified the DNA aptamer (VZ23) that can effectively bind, with low nanomolar affinity, to the extracellular domain of human FGFR1. In cells, VZ23 inhibited rat, mouse, and human FGFR1 signaling (Figures 1, S10, and S11; Table S6). This is not surprising given the high sequence conservation of the FGFR1 extracellular domain (Figure S12). VZ23 does not bind to the extracellular domains of human FGFR2, FGFR3, or FGFR4 and consequently does not inhibit signaling of FGFR2b, FGFR2c, FGFR3b, FGFR3c, and FGFR4 (Figures 1D and S10). Finally, the inhibitory effect on FGFR1 signaling was not limited to rat chondrocytes expressing FGFR1 as the sole FGFR, but was also observed in cultured mouse adipocytes, mesenchymal cells, fibroblasts and human embryonic kidney cells (Figure S11). Our data suggest that the VZ23 binding site on FGFR1 is distinct from the FGF binding site and that the inhibitory effect of VZ23 is FGF-independent (Figure 4G). How the binding of VZ23 blocks the transmission of allosteric signals from the extracellular FGF-induced FGFR1 dimer to the intracellular part of FGFR is the subject of ongoing structural biology studies. Furthermore, we showed that VZ23’s efficiency in inhibiting FGFR1 signaling positively correlates with its capacity to adopt an antiparallel G-quadruplex structure and its thermodynamic stability (Figure 2). This observation raises the possibility of further enhancing VZ23 efficiency through covalent modifications to increase the thermodynamic stability of the VZ23 scaffold.

Impaired FGFR1 signaling is associated with more than 10 different cancer diagnoses. Human craniosynostoses, characterized by premature fusion of the cranial sutures in early childhood, are also caused by mutations in FGFR1, including Pfeiffer syndrome and osteoglophonic dysplasia.^7^ FGFR TKIs, which represent the current state of the art in FGFR inhibitors, typically exhibit pan-FGFR activity and therefore do not provide targeted control of FGF signaling within an organ or tissue, which often involves several different FGF:FGFR interactions that regulate different cell types with various outcomes. The lack of tools to target individual FGFR variants hinders not only the understanding of FGF function but also progress in the treatment of FGFR-related diseases. While the utilization of the VZ23 scaffold as a therapeutic will necessitate further development, VZ23 can have immediate applications in molecular and cellular biology for addressing research questions that require isolating FGFR1 signaling from signaling originating from other FGFRs, as well as in biotechnology. Additionally, with its low cost, long shelf life/stability, and the potential for covalent modification, such as with fluorophores, VZ23 could emerge as a viable alternative to antibodies for use as a specific staining agent or as the foundation of FGFR1-specific affinity matrices.

## Materials and methods

### DNA oligonucleotides, SELEX, PCR, and aptamer cloning

A single-strand DNA (ssDNA) library comprising 10^15^ random 40-nt sequences flanked with primer binding sites (5′ATACCAGCTTATTCAATT-N40-AGATAGTAAGTGCAATCT3′) was synthesized in Ella Biotech, Germany. For SELEX, the recombinant human FGFR1c-Fc (RnD Systems) was immobilized on protein G magnetic beads (Thermo Fisher). The ssDNA library was preincubated with protein G magnetic beads and then incubated with the immobilized FGFR1c-Fc. Beads were washed with the binding buffer (20 mM Tris-HCl pH 7.6, 100 mM NaCl, 5 mM KCl, 2 mM MgCl_2_, 1 mM CaCl_2_), and bound aptamers were eluted, isolated by sodium acetate/ethanol precipitation, and amplified in subsequent PCR. In the fifth and sixth SELEX rounds, additional negative selection steps with recombinant cMet-Fc-coated beads (RnD Systems) were introduced. Table S2 summarizes SELEX conditions. Aptamers isolated in the sixth SELEX round were amplified by PCR using non-modified forward and reverse primers (Generi Biotech). PCR products were plasmid cloned using CloneJET PCR Cloning Kit (Thermo Fisher) and Sanger sequenced. Table S3 lists DNA sequences used in the study. The isotopically unlabeled VZ23 oligonucleotide used for 2D NOESY experiment and 10% residue-specific ^15^N/^13^C-labeled VZ23 oligonucleotides were synthesized on DNA/RNA H-8 Synthesizer (K&A Laborgeräte GbR) using standard phosphoramidite chemistry with dimethyltrityl protecting group. Oligonucleotides were cleaved from the solid support and deprotected with ammonium hydroxide and methylamine in a 1:1 (v/v) ratio for 30 min at room temperature and 30 min at 65°C. Samples were purified using GlenPak cartridges (Glen Research) and desalted on fast protein liquid chromatography with HiPrep 26/10 Desalting column (GE Healthcare). Samples were dried using a vacuum centrifuge and afterward dissolved in 300 μL of deionized water. Concentrations were determined by UV absorption at 260 nm using a Varian Cary 100 Bio UV/VIS spectrophotometer. The extinction coefficients were determined by the nearest neighbor method.

### Quantitative RT-PCR

A total of 3 × 10^5^ cells were plated in a six-well plate for 24 h. RNA was isolated using RNA blue reagent (Top-Bio) and cDNA was synthesized using oligo-dT primers from 1 μg of total RNA using Revertaid H minus First-strand cDNA synthesis kit (Thermo Fisher). Table S4 lists used PCR primers. PCR reactions were performed in technical triplicates using Lightcycler 480 SYBR Green Master I kit (Roche). Annealing temperature was 58°C. The amount of transcript was measured as the threshold cycle (Ct) and normalized to *Ubb* (ΔCt). ΔΔCt was calculated by comparing the expression of each gene with the RCS *null* cell. The final fold change of expression is represented by 2ˆ-ΔΔCt. Statistical significance (unpaired t test) was calculated using GraphPad Prism 5.

### NMR and CD spectroscopy

NMR spectra were measured on Bruker Avance *NEO* spectrometers (Bruker) at 600, 850, or 950 MHz using triple-resonance 5-mm cryogenic probes. Spectra of 50-μM DNA samples in the PBS (8.1 mM Na_2_HPO_4_, 1.5 mM NaH_2_PO_4_, 137 mM NaCl, 2.7 mM KCl), binding buffer, or 10% DMEM and 10% D_2_O were recorded at 20 or 37°C. One-dimensional ^1^H NMR spectra were acquired using a 3-9-19 pulse sequence with gradients to suppress the water signal. Spectra were baseline corrected and processed with the exponential apodization function with the line-broadening parameter set to 10 MHz. Imino protons were assigned using ^15^N-edited HSQC spectra, while imino-aromatic and anomeric-aromatic NOE connections were identified using ^1^H–^1^H 2D NOESY spectra with 250 ms mixing time. The spectra were acquired and processed with TopSpin v4.3.0 (Bruker, Germany). One-dimensional ^1^H and ^15^N-edited NMR spectra were analyzed using MestReNova v14.2.2 or v14.3.3 (Mestrelab Research), while the resonances on 2D NOESY spectra were assigned using NMRFAM-Sparky software (UCSF). CD measurements were acquired using Jasco J815 spectropolarimeter (Jasco). Aptamers were refolded before each measurement. CD spectra were obtained at λ220-320 nm. CD melting experiments were carried out in the 4°C–94°C range at 2°C/min heating rate, with changes in the signal monitored at λ 289 nm. The UV absorption spectra for TDS analysis were acquired simultaneously with CD experiments on Jasco J815 spectropolarimeter. Data were processed and plotted using GraphPad Prism 8 software.

### Recombinant FGFR production and BLI

Fully glycosylated extracellular domains of human FGFR1b, FGFR1c, FGFR2b, FGFR2c, FGFR3b, FGFR3c, and FGFR4 were fused to the Fc domain of IgG1 and purified as described before.^17^ BLI experiments were performed on Octet K2 (Sartorius AG) using protein A biosensors. FGFRs were immobilized on a protein A biosensor at a 5 μg/mL concentration and incubated with VZ23 serially diluted in PBS or PBS supplemented with 2 mM MgCl_2_ at concentrations ranging from 31.25 to 500 nM. Association and dissociation steps were monitored for 180 s each. Equilibrium dissociation constants (K_D_) were calculated using a global fitting with a 1:1 model and steady-state analysis. For BLI competitive binding experiments, FGFR1c or FGFR1b were immobilized on protein A biosensors at a concentration of 5 μg/mL, and measurements were conducted with VZ23 and FGF1 diluted in PBS to 250 nM each. The first association step was measured for VZ23 only and the second for an equimolar mixture of VZ23 and FGF1 followed by dissociation in PBS. Alternatively, a second set of measurements was performed with the first association measured for FGF1 only, repeating the other steps. All stages were monitored for 180 s each. The data obtained were analyzed using Data Analysis 11.0 software from ForteBio.

### Cell culture, western blot, and KroxDS reporter assay

Cells were propagated in DMEM, supplemented with 10% FBS and antibiotics (Invitrogen). 3T3-L1 cells were differentiated by 3 days’ induction with DMEM supplemented with 10% FBS, 0.5 mM isobutylmethylxanthine, 1 μM dexamethasone (Sigma), and 1 μg/mL insulin (Tocris) and maturated in DMEM supplemented with 10% FBS and 1 μg/mL of insulin until day 12. Human recombinant FGF1, FGF2, FGF4, SCF, insulin, HGF and EGF were from Biotechne. Heparin (1 μg/μL; Sigma) was used in FGF treatment experiments. RCS cells expressing endogenous single FGFR2c, FGFR3c, or FGFR4 were generated from wild-type RCS cells where endogenous *Fgfr1-4* genes were individually targeted by CRISPR-Cas9 to leave only one single FGFR expressed. The RCS cells expressing endogenous-only FGFR1c (RCS-FGFR1c) were prepared similarly, using the *Fgfr3/Fgfr4* double-knockout RCS cells (a gift from Carmine Settembre, Telethon Institute of Genetics and Medicine, Pozzuoli, Italy). The RCS FGFR1-4 *null* cells were generated from RCS cells where all four endogenous *Fgfr* genes were targeted by CRISPR-Cas9. The cells expressing a single human FGFR variant (FGFR1b, FGFR1c, FGFR2b, FGFR2c, FGFR3b, FGFR3c, or FGFR3b) were generated by stable transfection of FGFR1-4 *null* cells with vectors containing the individual V5-tagged, full-length human *FGFR1-4*. A stable integration was achieved by PiggyBac transposase and low FGFR expression was ensured by attenuated cytomegalovirus promotor, as described earlier.^12^ KIT (Addgene_201986; addgene.org/201986), FGFR1c (Addgene_201106; addgene.org/201106), and other FGFR expression vectors were described before.^23^ The pKrox24(MapERK)d1EGFP (KroxDS) reporter (Addgene_214912; addgene.org/214912) was generated from pKrox24(MapERK)dsRed reporter (Addgene_200114; addgene.org/200114), by replacing the dsRed with destabilized d1EGFP, supplemented with 3′UTR of the mouse *Egr1* gene. The 12XCSL-d1EGFP vector was a gift from Urban Lendahl (Addgene_47684; addgene.org/47684).^26^ KroxDS was stably integrated into RCS-FGFR1c cells by PiggyBac transposase; transactivation was determined by automated microscopy (Nikon BioStation). For FGF1 secretion, U2OS cells stably transfected with empty pcDNA3.1 vector (U2OS-pcDNA3.1) and pcDNA3.1 vector encoding human FGF1 C-terminally tagged with Myc (U2OS-FGF1myc) were prepared as described previously.^27^ To induce FGF1 secretion, serum-starved cells were incubated at 42°C for 2 h. The FGF1-conditioned media was collected, centrifuged, and concentrated on an Amicon filter (Sigma). Conditioned media from U2OS cells transfected with an empty pcDNA3.1 was used as a control. NIH3T3 cells were incubated with VZ23 for 30 min, followed by a 15 min incubation with media conditioned by U2OS cells for 15 min in the presence of heparin (10 U/mL). For western blot, cells were harvested directly into the Laemmli sample buffer; lysates were resolved by SDS-PAGE, transferred onto a PVDF membrane, and visualized by chemiluminescence (Thermo Fisher). Western blot signal was quantified in ImageJ (http://imagej.nih.gov/ij/). Table S5 lists all used antibodies.

### Statistical analyses

Unless otherwise indicated, all experiments were performed at least in triplicate; the *n*-value states the number of independent experiments and is shown in each figure panel. In the bar and line graphs, data are expressed as mean ± SEM or mean ± SD. The statistical method used to analyze the data is indicated in each figure.

## Data and code availability

All data are incorporated into the article and its online supplemental material. Raw data are available on request.

## Acknowledgments

We thank Carmine Settembre for FGFR3/4 double-knockout RCS cells. We thank projects that provide access to research infrastructure: we acknowledge CF Josef Dadok NMR Center and the CF Biomolecular Interactions and Crystallography of CIISB, Instruct-CZ Center, supported by 10.13039/501100001823MEYS CR (LM2023042), the 10.13039/501100008530European Regional Development Fund - Project “UP CIISB” (No. CZ.02.1.01/0.0/0.0/18_046/0015974) and 10.13039/100020931CERIC-ERIC consortium (No. 20242221). This work was supported by the National Institute for Cancer Research Programme EXCELES, ID Project No. LX22NPO5102 - co-financed by the European Union - Next Generation EU; Ministry of Education, Youth and Sports of the Czech Republic (LUAUS23295); Agency for Healthcare Research of the Czech Republic (NU21-06-00512, NU23-10-00550); 10.13039/501100001824Czech Science Foundation (GF21-26400K); Grant Agency of Masaryk University (MUNI/G/1771/2020); M.L.Z. acknowledges support from 10.13039/501100004329Slovenian Research And Innovation Agency (P1-0242); P.K. is supported by Praemium Academiae of the Czech Academy of Sciences. G.R.-L. is supported by a postdoctoral fellowship of the Internal Grant Agency of the Faculty of Medicine, 10.13039/501100010653Masaryk University. Figures 2A, S8A, and graphical abstract were created with Bio-Render (biorender.com).

## Author contributions

V.Z., Z.F., P.K., and L.T. developed VZ23; Z.F., A.C., B.F., V.-C.U., P.D., G.R.-L., V.R., A.K., T.R., K.S., and M.S. performed cell experiments; J.C., M.B., D.K., M.Z., V.Z., J.R., S.F.-T., M.L.Z., and M.K. performed biophysical experiments; M.Z., K.H., L.T., and P.K. designed research; all authors analyzed data and interpreted experiments; L.T. and P.K. wrote the article with contributions from all authors; and L.T. and P.K. provided funding. All authors have approved the final version of the manuscript.

## Declaration of interests

The authors declare no conflicts of interest.

## Supplemental information

Document S1. Figures S1–S12 and Tables S1–S6

Document S2. Article plus supplemental information
