# Supplementary material for: Specific inhibition of fibroblast growth factor receptor 1 signaling by a DNA aptamer
Source: Mol Ther Nucleic Acids. 2024 Nov 28;36(1):102405. doi: 10.1016/j.omtn.2024.102405 (PMC11700292; doi:10.1016/j.omtn.2024.102405)
Supplement: Document S1. Figures S1–S12 and Tables S1–S6 [file mmc1.pdf]

OMTN, Volume *36*

Supplemental information

Specific inhibition of fibroblast growth factor receptor 1 signaling by a DNA aptamer

Vladimira Zlinska, Zuzana Feketova, Aleksandra Czyrek, Julia Chudzian, Martina Lenarcic Zivkovic, Vlad-Constantin Ursachi, Pooja Dudeja, Bohumil Fafilek, Jan Rynes, Gustavo Rico-Llanos, Adolf Koudelka, Tanaya Roy, Martyna Biadun, Vendula Raskova, Katerina Svozilova, Michaela Stroblova, Mateusz Krzyscik, Kalina Hristova, Daniel Krowarsch, Silvie Foldynova-Trantirkova, Malgorzata Zakrzewska, Lukas Trantirek, and Pavel Krejci

Table S1 FGFR TKIs currently evaluated in cancer clinical trials (clinicaltrials.gov*; medchemexpress.com¶)

TKI (IC_{50}, nM)	*Clinical trials**	*FGFR1*	*FGFR2*	*FGFR3*	*FGFR4*	*Other inhibited kinases*	*Reference*
Infigratinib	20+	0.9	1.4	1	60	VEGFR2, cKIT	1
Pemigatinib	30+	0.4	0.5	1	30	VEGFR2, cKIT	2
Lenvatinib	20+	46	-	-	-	VEGFR1-3, PDGFRa/b, cKIT	¶
Erdafitinib	15+	1.2	2.5	3	5.7	RET, CSF1R, PDGFRa, VEGFR3, cKIT	3, ¶
AZD4547	10+	0.2	2.5	1.8	165	VEGFR2, IGF1R	4, ¶
Futibatinib	15+	1.8	1.4	1.6	3.7	-	5
Derazantinib	10+	4.5	1.8	4.5	34	RET, DDR2, FMS, PDGFRa/b, VEGFR1-3	6
Rogaratinib	8	11.2	1	18.5	201	VEGFR3	¶
Lucitanib	7	17.5	82.5	237.5	>1000	cKIT, CSF1R, PDGFRa/b, VEGFR1-3	7, ¶
E7090	6	0.71	0.5	1.2	120	RET, DDR2, VEGFR1-3, PDGFRa/b	8
Gunagratinib	5	-	-	-	-	-	¶
Zoligratinib	3	9.3	7.6	22	290	PDGFRb	9
LY2874455	2	2.8	2.6	6.4	6	VEGFR2	10, ¶
RLY-4008	1	-	-	-	-	MEK5, MKNK2	11
Roblitinib	1	>10mM	>10mM	>10mM	6.2	-	12

Table S2 SELEX conditions

		Positive selection				*Negative selection*	
Round	*Input ssDNA (pmol)*	*FGFR1c-Fc (pmol)*	*Incubation volume (µl)*	*Incubation time (min)*	*Number of washes*	*Protein G magnetic beads*	*cMET-Fc (pmol)*
1	2000	100	500	30	3	126 µL	-
2	100	40	500	30	3	20 µl×5	-
3	100	40	500	30	3	20 µl×5	-
4	30	40	500	30	3	20 µl×5	-
5	5	2	1000	10	3×5 min	20 µl×3	3×5
6	5	2	1000	10	3×5 min	20 µl×3	3×5

Table S3 DNA oligonucleotides used in the study. SELEX primer sequences are highlighted in gray.

Name	*Sequence (5'to 3')*
Forward	ATACCAGCTTATTCAATT
Reverse	(biotin)AGATTGCACTTACTATCT
A	ATACCAGCTTATTCAATTGGCAGGGGATGGTGTGGTTATTTGATACTGGTAGAAGGCAAGATAGTAAGTGCAATCT
B	ATACCAGCTTATTCAATTACTACGGCAGTGATTTGGCAGGGGATGGTGTGGTCCGGCTAGATAGTAAGTGCAATCT
C	ATACCAGCTTATTCAATTTACGATATTTATGGTCTGGATGGTCGGTTATAGTAATTCGAGATAGTAAGTGCAATCT
D	ATACCAGCTTATTCAATTTTGGTGTGGATGGGGTAGGGACTTTAGTAAATTGAAAGGGAGATAGTAAGTGCAATCT
E	ATACCAGCTTATTCAATTCGCCCGTGGAGTGGAGGGCAGGGGCTCGGTTCTTTTAGCGAGATAGTAAGTGCAATCT
F	ATACCAGCTTATTCAATTGGTGTGGATGGCAGGGGTCATTTGTATAGCGGATTATAATAGATAGTAAGTGCAATCT
G	ATACCAGCTTATTCAATTGATTTCCTATCTGAGTGGTGTGGATGGCAGGGGCTTGAGAAGATAGTAAGTGCAATCT
H	ATACCAGCTTATTCAATTGTACGGTCACGTTGGTGTGGATGGCAGGGGAGTGACAGAGAGATAGTAAGTGCAATCT
I	ATACCAGCTTATTCAATTGGGATACAGGGCTTTGTCTATGGTGTGGATGGCGGATACCAGATAGTAAGTGCAATCT
J	ATACCAGCTTATTCAATTGTGGTATGTGGGCTCTATCGATGGTGTGGATGGCAGGGGCAGATAGTAAGTGCAATCT
VZ23	GGGATACAGGGCTTTGTCTATGGTGTGGATGGCGGATACC
Scramble	GCTGTCGGTTGCGTGCGTGTGATGAGAAGATGCCGTTAAG
Reverse	CCATAGGCGGTAGGTGTGGTATCTGTTTCGGGACATAGGG
VZ23-T14A	GGGATACAGGGCTATGTCTATGGTGTGGATGGCGGATACC
VZ23-G2T	GTGATACAGGGCTTTGTCTATGGTGTGGATGGCGGATACC
VZ23-G25T	GGGATACAGGGCTTTGTCTATGGTTTGGATGGCGGATACC

..

Table S4 Quantitative RT-PCR primers used in the study

Gene	*Primer*	*Sequence (5' to 3')*
Ubb	Forward	ATGTGAAGGCCAAGATCCAG
	Reverse	TAATAGCCACCCCTCAGACG
Fgfr1	Forward	CTCTGTGGTGCCTTCTGACA
	Reverse	TTCACCTCGATGTGCTTCAG
Fgfr2	Forward	ACTGGACCAACACCGAAAAG
	Reverse	CTCCACCAGGCAGGTGTAAT
Fgfr3	Forward	GGGCTTCTTCCTCTTCATCC
	Reverse	GGACGAGAGGTGTGTTGGAG
Fgfr4	Forward	TCGATCCACTTTGGGAGTTC
	Reverse	CAGGTCTGCCAAATCCTTGT

Table S5 Antibodies used in the study

Protein	*Source*	*Cat. No.*
Actin	Cell Signaling	3700
AKT	Cell Signaling	4691
$pAKT^{S473}$	Cell Signaling	4060
Caveolin 1	Cell Signaling	3238
Collagen 2	Abcam	ab34712
ERK	Cell Signaling	9102
$pERK^{T202/Y204}$	Cell Signaling	9101
FGF1	Santa Cruz Biotechnology	sc-55520
FGFR1	Cell Signaling	9740
FGFR2	Cell Signaling	23328
FGFR3	Cell Signaling	4574
$pFGFR1^{Y653/Y654}$	Sigma	06-1433
$pFRS2^{Y196}$	Cell Signaling	3864
GAB1	Cell Signaling	3232
$pGAB1^{Y627}$	Cell Signaling	3231
GFP	Abcam	ab290
INSR	Cell Signaling	3025
$pINSR^{Y1150/Y1151}$	Cell Signaling	3024
Lamin A/C	Cell Signaling	2032
Tubulin	Abcam	ab11316
Vinculin	Cell Signaling	13901
V5	Invitrogen	46-0705
mouse IgG	Sigma Aldrich	A6782
rabbit IgG	Sigma Aldrich	A6667

Table S6 Species of the FGFRs analyzed in the study

Figure	*FGFR isoform (species)*
1A	1c (rat)
1B	1b, 1c (rat)
1C	1b, 1c, 2b, 2c, 3b, 3c, 4 (human)
1D	1b, 2b, 3b (h); 2c, 3c, 4 (rat)
3B	1c (rat)
4B-F	1c (rat)
4G	1c (human)
S1A	1c, 2c, 3c (rat)
S1B	1, 2, 3, 4 (rat)
S1C	1b, 1c, 2b, 2c, 3b, 3c, 4 (human)
S2	1c (rat)
S3A	1c (rat)
S3B	1c (rat)
S3C	1c, 1b (human)
S3D	1c (rat), 1b (human)
S5C, D	1c (human)
S7A	1c (rat)
S7B	1b (human)
S8B	1c (human)
S9	1c, 2c, 3c, 4 (rat)
S10	1c, 2c, 3c, 4 (human)
S11A	1c (mouse)
S11B	1c (mouse)
S11C	1c (human)
S11D	1c (mouse)

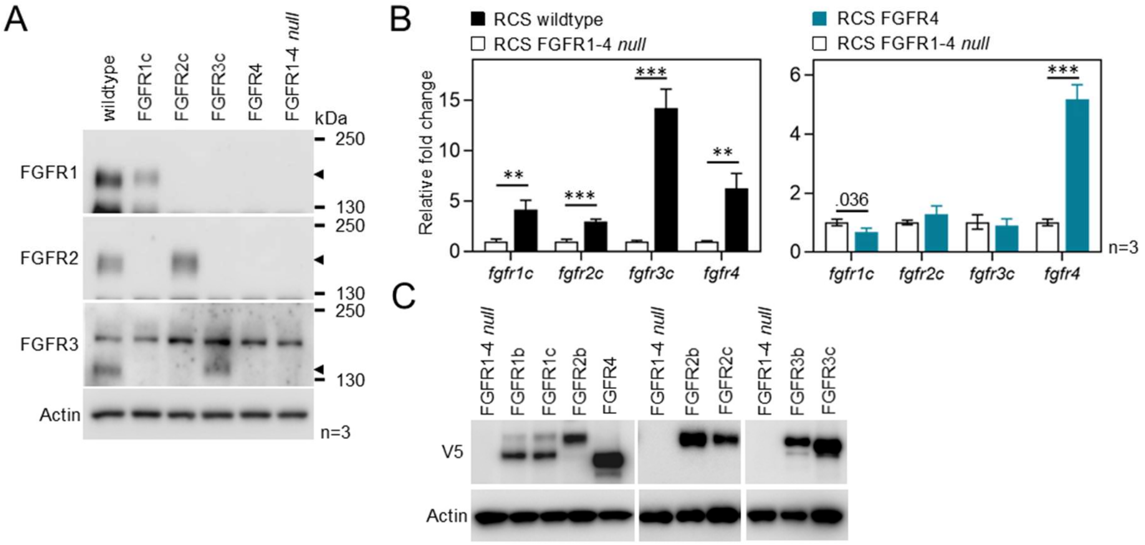

Figure S1 FGFR1-4 expression in the cell models used. (**A**, **B**) Wildtype RCS cells express endogenous rat FGFR1c, FGFR2c, FGFR3c and FGFR4. CRISPR-Cas9 was used to inactivate individual FGFR genes to generate cell lines expressing a single FGFR variant. *Null*, RCS cells with inactivated endogenous *fgfr1-4* genes. (**B**) Expression of *fgfr1-4* in RCS cells was monitored by quantitative (q) RT-PCR. The mRNA levels were normalized to RCS *null* cells for each gene (mean±SD; t-test, **p<0.01, ***p<0.001; n, number of independent experiments). (**C**) RCS cells *null* for endogenous FGFR1-4 were stably transfected with V5-tagged human FGFR variants. Western blots show the expression levels of the respective FGFR isoforms; actin was used as a loading control.

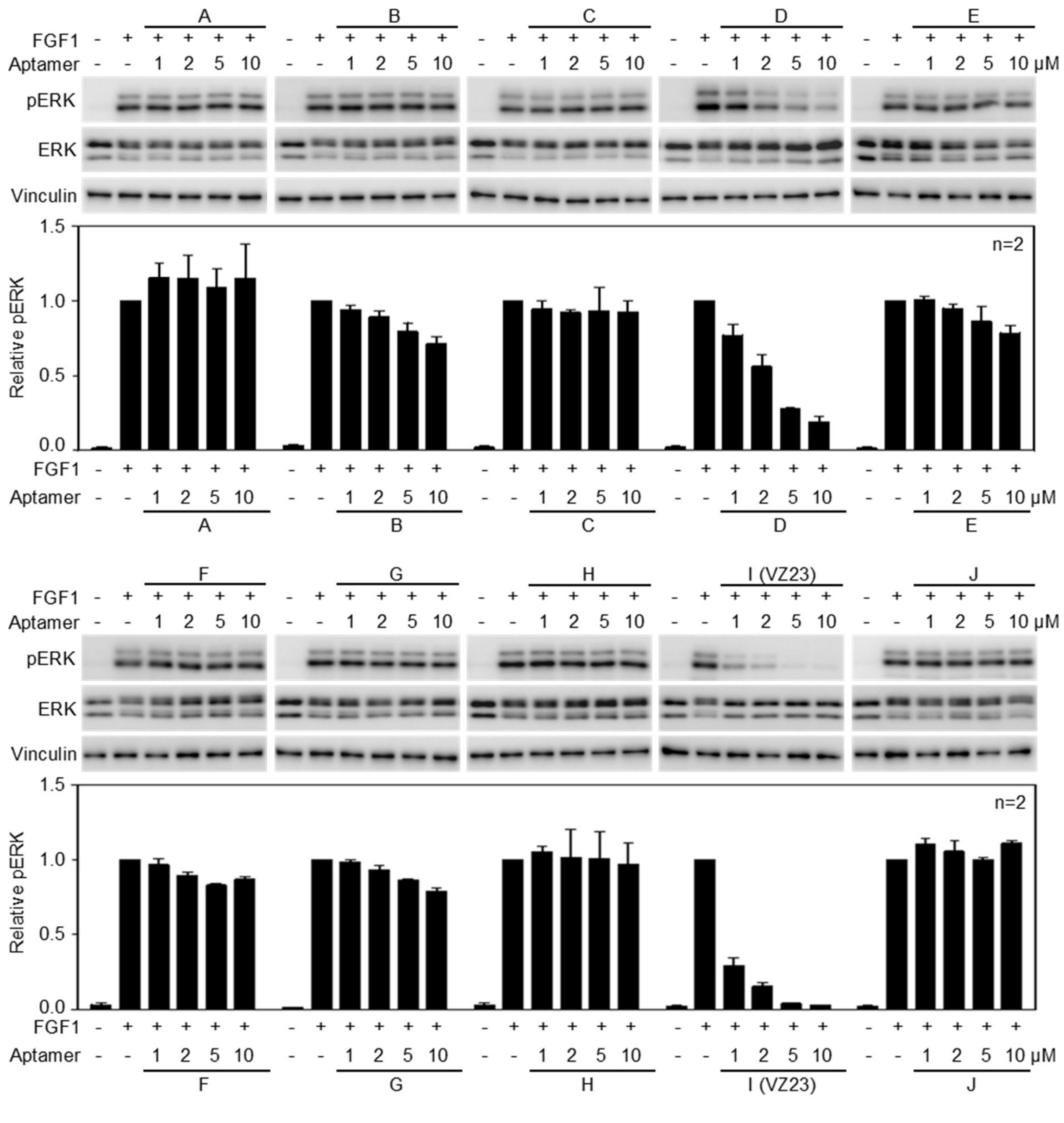

Figure S2 RCS-FGFR1c (rat) cells were treated with SELEX aptamers A-J for 30 minutes, followed by heparin (1 µg/ml) and FGF1 (5 ng/ml) treatment for 1 hour. FGF1-mediated phosphorylation (p) of ERK MAP kinase was determined by western blot. Total ERK and vinculin served as loading controls. Western blot data were quantified and graphed (mean±SD; n, number of independent experiments). Aptamers D and I show an inhibitory effect on FGF1-mediated ERK activation. Aptamer I was designated VZ23 and further characterized.

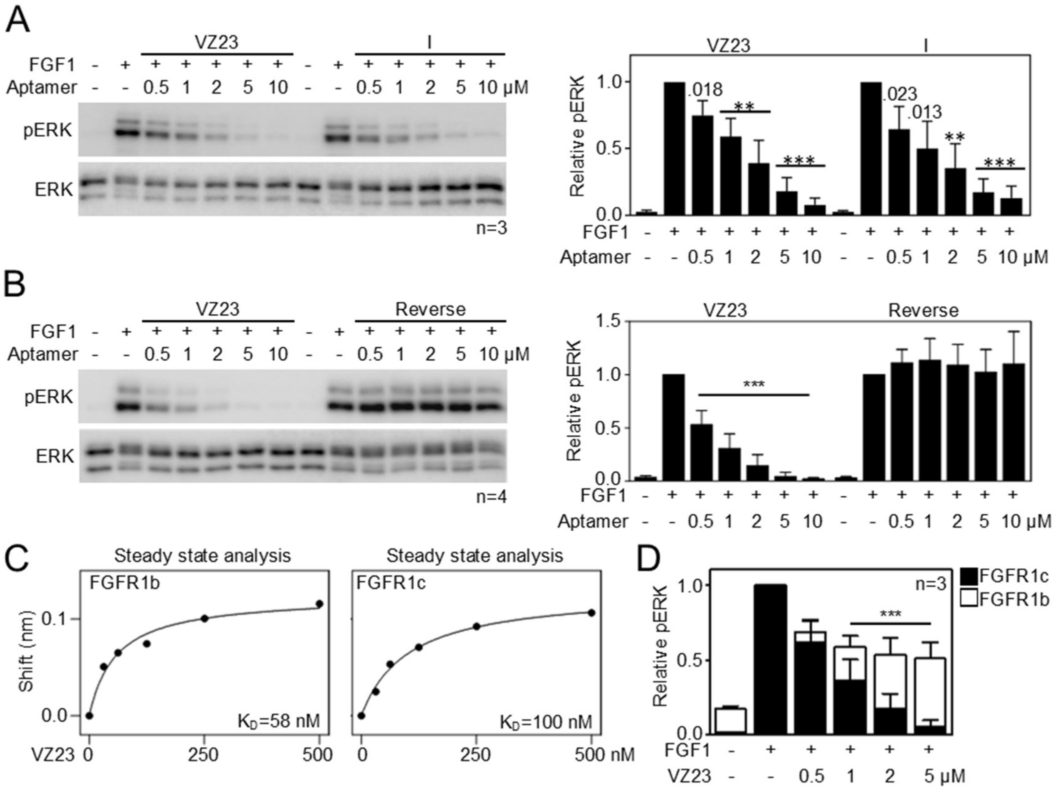

Figure S3 (**A**) Comparison of VZ23 and Aptamer I (VZ23 with SELEX primers). RCS-FGFR1c (rat) cells were treated with aptamers for 30 minutes followed by heparin (1 µg/ml) and FGF1 (5 ng/ml) treatment for 1h. Cells were analyzed for fosforylated (p) ERK activation by western blot. Total ERK serves as a loading control. pERK signal was quantified and plotted (mean±SD; t-test, **p<0.01, ***p<0.001; n, number of independent experiments). (**B**) RCS-FGFR1c cells were treated with VZ23 or reverse aptamer. The FGF1-mediated phosphorylation (p) of ERK MAP kinase was determined by western blot. Total ERK served as loading control. Western blot signal was quantified and graphed (mean±SD; ANOVA, *** p<0.001; n, number of independent experiments). VZ23 inhibits FGF1-mediated activation of ERK, reverse aptamer has no effect. (**C**) Steady-state analysis for representative binding profiles of VZ23 to human FGFR1b and FGFR1c. Kinetic parameters were determined by global fitting with the 1:1 model and steady-state analysis. (**D**) Comparison of the VZ23 effect on FGF1-mediated ERK activation in RCS-FGFR1b (human) and RCS-FGFR1c (rat) cells (quantification of the western blot data shown in Fig. 1A, D; two-way ANOVA).

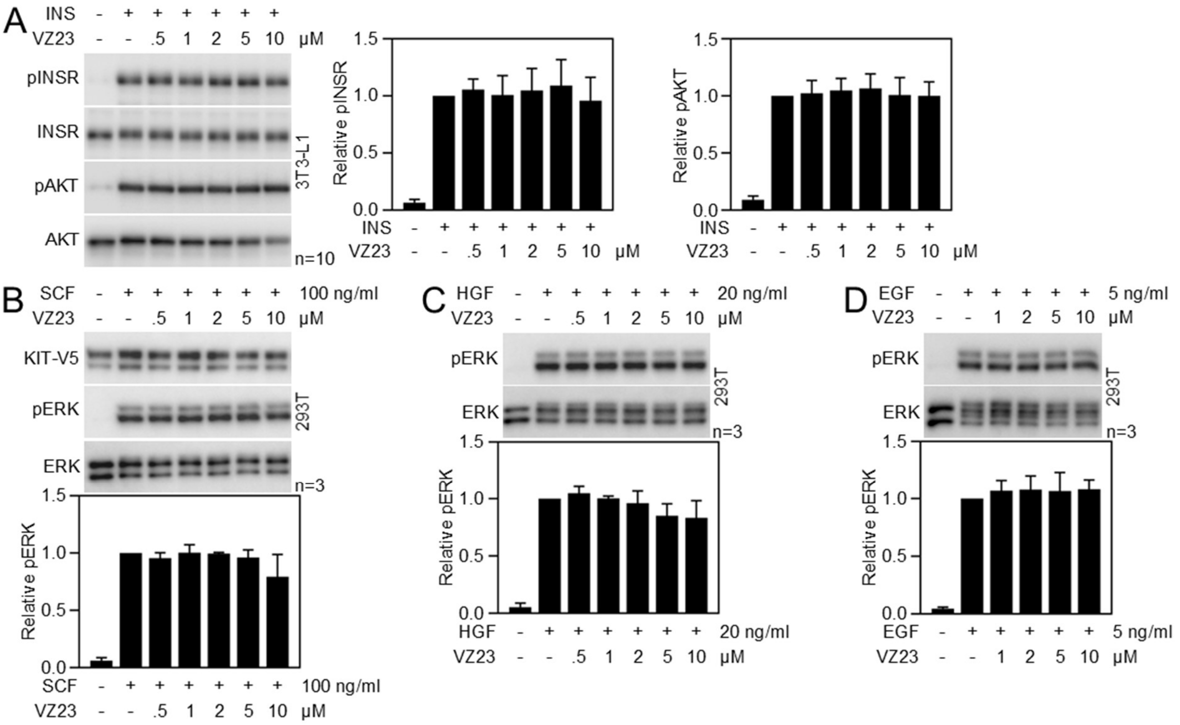

Figure S4 VZ23 does not interfere with INSR, MET, KIT and EGFR signaling. (**A**) 3T3-L1 cells were differentiated into adipocytes and incubated with VZ23 for 30 min, then activated with 0.1-1 µM of recombinant insulin (INS) for 1 hour and immunoblotted for phosphorylated (p) INSR and AKT. (**B**) 293T cells were transfected with the V5-tagged KIT according to the FuGENE6 protocol, incubated with 0.5-10 µM VZ23 for 30 min, then activated with 100 ng/ml of recombinant KIT ligand SCF for 30 min and immunoblotted for ERK activity; V5 was used as a transfection control. (**C**, **D**) 293T cells were incubated with 0.5-10 µM VZ23 for 30 min, then activated with 20 ng/ml of recombinant MET ligand HGF or 5 ng/ml of recombinant EGFR ligand EGF for 30 min and immunoblotted for phosphorylated (p) INSR, ERK or AKT, the signal was quantified and plotted (mean±SD; n, number of independent experiments).

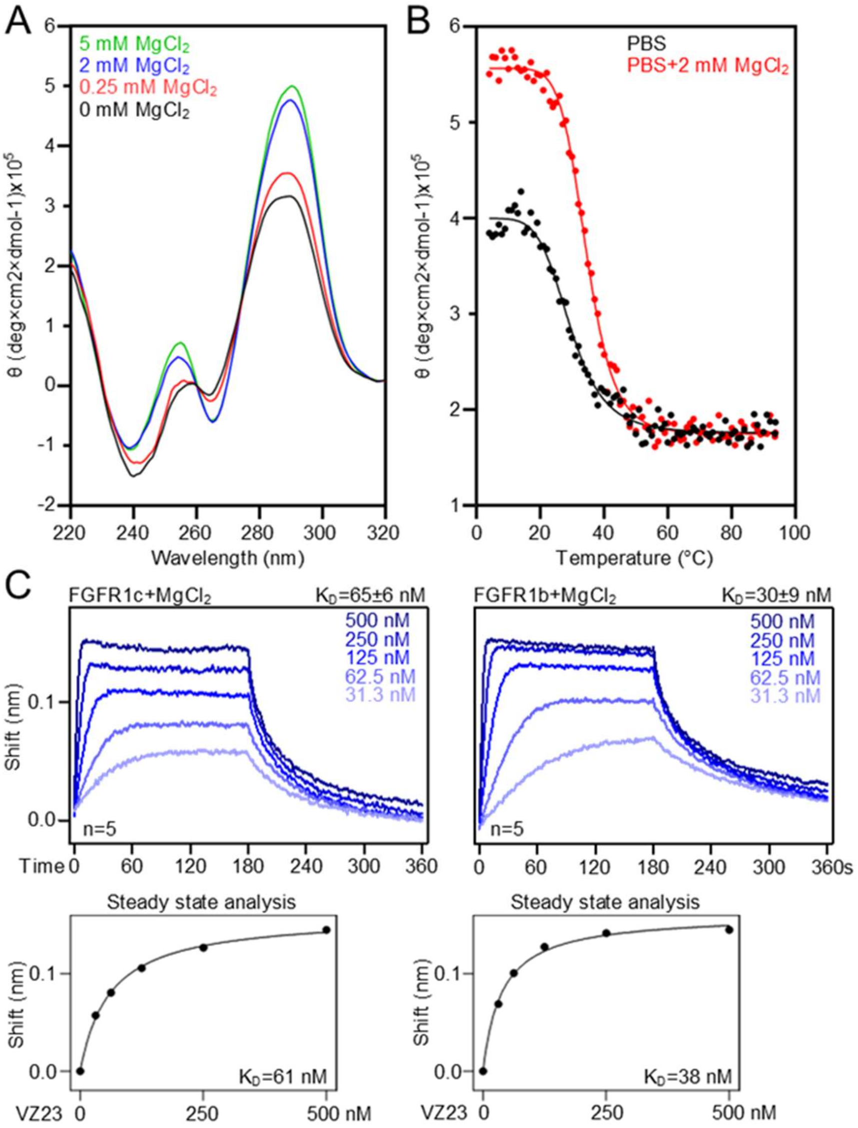

D

Exp.	FGFR1c	FGFR1c 2 mM $MgCl_2$	FGFR1b	FGFR1b 2 mM $MgCl_2$
1	100 nM	61 nM	58 nM	38 nM
2	190 nM	69 nM	55 nM	41 nM
3	190 nM	58 nM	61 nM	24 nM
4	210 nM	70 nM	52 nM	24 nM
5	120 nM	69 nM	47 nM	22 nM
$\bar{x}$	162±48.68 nM	65.4±5.5 nM	54.6±5.41 nM	29.8±8.96 nM

Figure S5 (**A**) CD spectra of VZ23 at 20°C in PBS pH 7.3 (black) titrated with $MgCl_2$ to 0.25 mM (red), 2 mM (blue), and 5 mM (green) final concentration. (**B**) CD melting profiles of VZ23 in PBS, pH 7.3 (black), and PBS supplemented with 2 mM $MgCl_2$ (red) detected at 289 nm. (**C**) Interaction of VZ23 aptamer to FGFR1c was measured in the presence of $MgCl_2$. Serial twofold dilutions (31.3 to 500 nM) of the VZ23 were analyzed for association and dissociation in the presence of 2 mM $MgCl_2$ with FGFR1c and FGFR1b immobilized on Protein A sensors (top graphs). The kinetic parameters of the interaction were determined based on global fitting with the 1:1 model and steady-state analysis (bottom graphs). (**D**) Data from five independent BLI experiments are shown

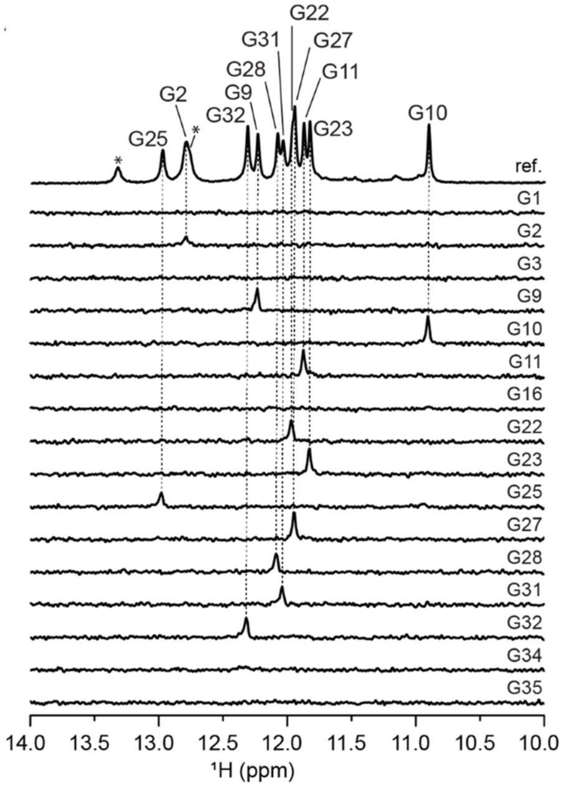

Figure S6 Unambiguous assignment of guanine imino proton resonances of VZ23 achieved by recording 1D ^{15}N-edited HSQC spectra on 10 % residue-specific ^{15}N/^{13}C-isotopically labeled oligonucleotides. Imino region of 1D ^{1}H NMR spectrum of VZ23 and assignment of resonances are shown on top. Imino resonances belonging to thymine residues, which probably form extra G-quartet structural elements, are marked with *.

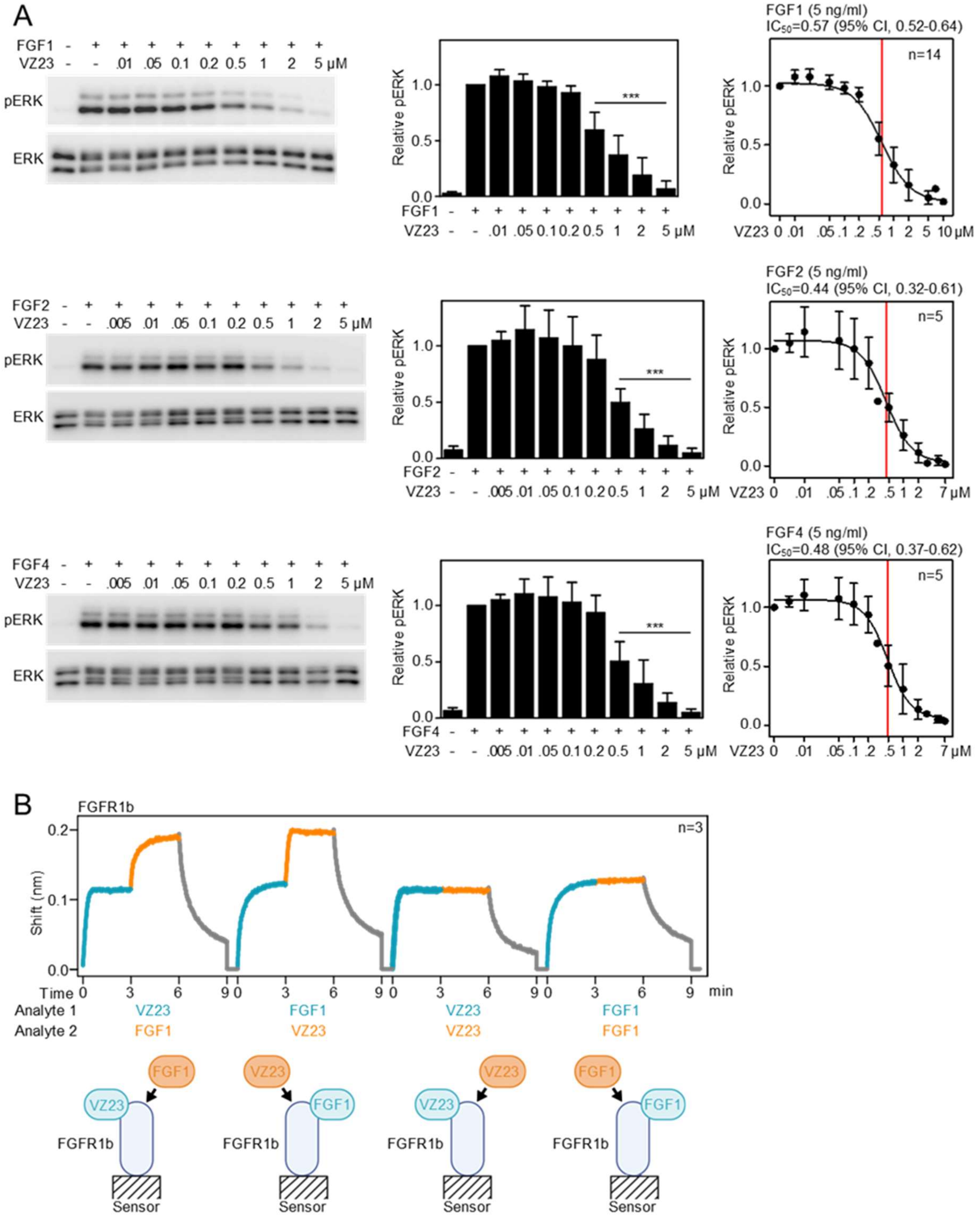

Figure S7 (**A**) The RCS-FGFR1c (rat) cells were treated with VZ23 and either FGF1, FGF2 or FGF4. Activation of ERK pathway was determined by western blot, quantified, graphed and the IC_{50} was calculated. Graphs show compilations of all experiments carried out; n, number of independent experiments. (**B**, **C**) BLI experiments to evaluate the binding of FGF1 to the already formed VZ23:FGFR1b (human) complex (**B**) or binding of VZ23 to the already formed FGF1:FGFR1b complex (**C**).

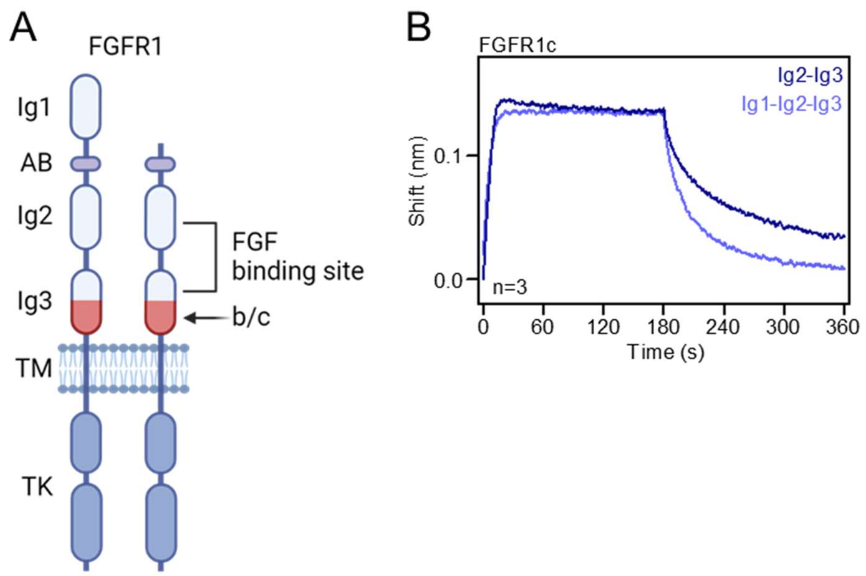

Figure S8 VZ23 binds to the truncated human FGFR1c extracellular domain, which lacks the N-terminal Ig-like domain 1 (Ig1). (**A**) Topology of the FGFR1 protein domains. Ig1, Ig2, Ig3, three extracellular Ig-like domains; AB, acid box; TM, transmembrane domain; TK, tyrosine kinase domain. The FGF binding site is indicated. Alternative splicing of the C-terminal part of Ig3 generates “b” or “c” variants of FGFR1. (**B**) BLI comparison of VZ23 binding to the full-length (Ig1-Ig2-Ig3) and truncated (Ig2-Ig3) extracellular domain of FGFR1, suggesting that the VZ23 binding site is not localized in the Ig1 domain (n, number of independent experiments).

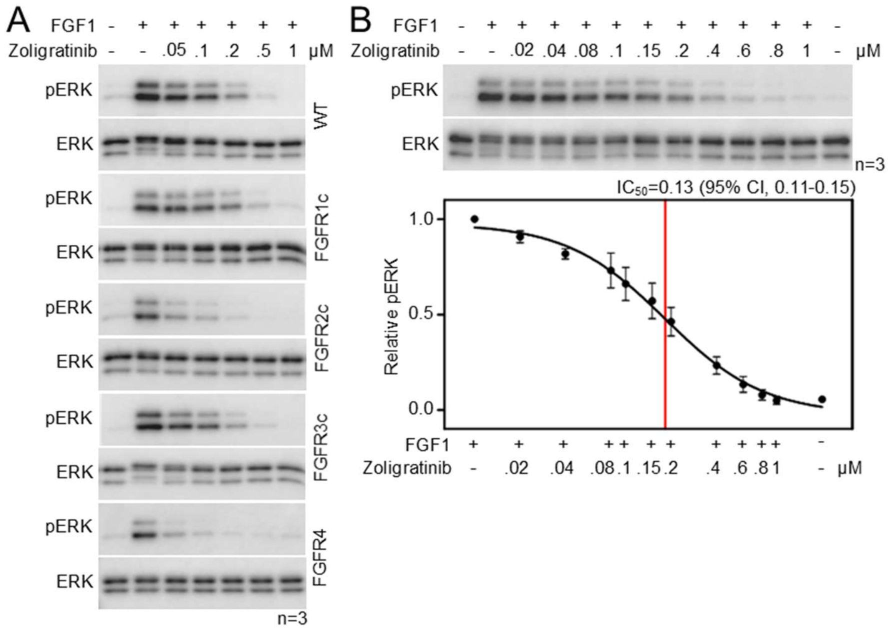

Figure S9 Effect of zoligratinib on FGFR signaling. (**A**) Wildtype RCS cells and RCS cells expressing endogenous FGFR1c, FGFR2c, FGFR3c and FGFR4 were treated with zoligratinib for 30 minutes before treatment with FGF1 for one hour and analyzed for phosphorylated (p) ERK by western blot; total ERK serves as a loading control. Zoligratinib shows no FGFR isoform selectivity as it inhibits signaling of all FGFR variants. (**B**) IC50 dose-response curve for the inhibitory effect of zoligratinib on FGFR1c-mediated activation of the ERK signaling pathway. Data from three independent experiments (n) were plotted (mean±SD).

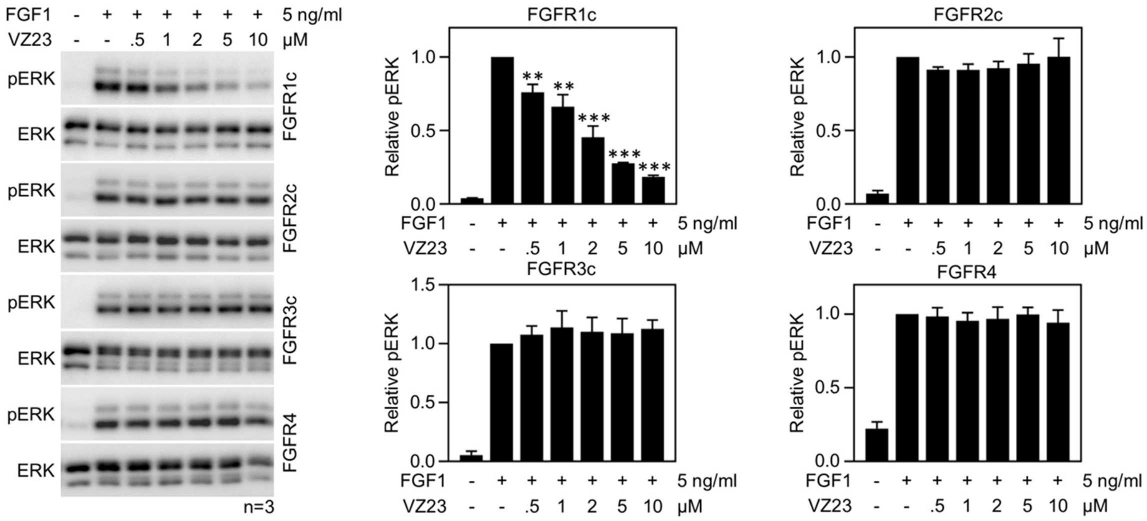

Figure S10 VZ23 inhibits signaling of human FGFR1c. RCS cells *null* for endogenous FGFR1-4 and stably expressing human FGFR1c, FGFR2c, FGFR3c or FGFR4 were treated with VZ23 for 30 minutes before FGF1 was added for 1 hour and assayed for phosphorylated (p) ERK. Total ERK serves as a loading control. The pERK signal was quantified and plotted (mean±SD; t-test, **p<0.01, ***p<0.001; n, number of independent experiments). Only FGFR1c-mediated activation of ERK pathway was inhibited by VZ23.

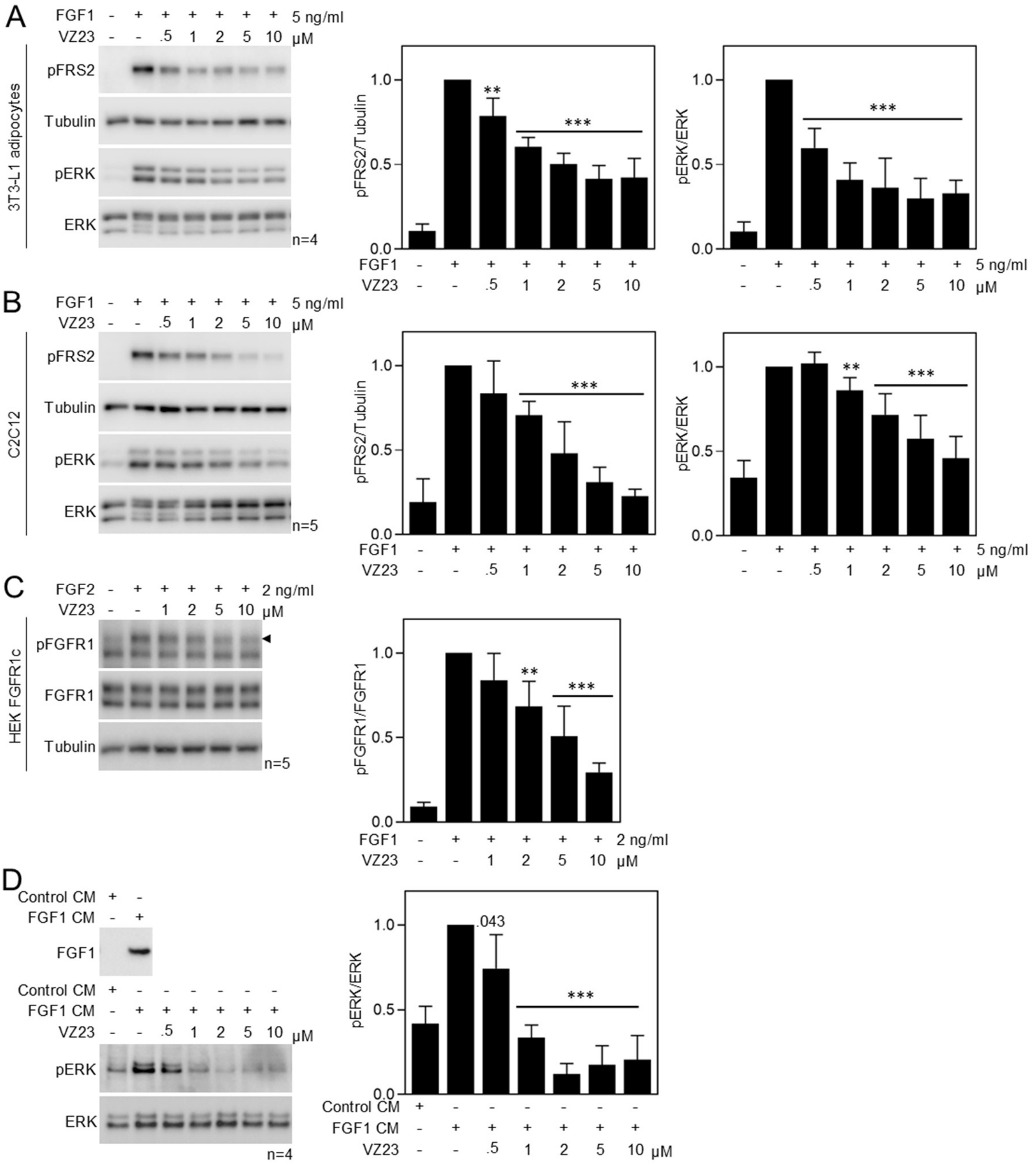

Figure S11 VZ23 inhibits FGFR1 signaling in (**A**) mouse 3T3-L1 adipocytes, (**B**) mouse C2C12 mesenchymal cells and (**C**) human embryonal kidney 293T cells expressing FGFR1c C-terminally tagged with V5. Cells were treated with VZ23 30 minutes before the addition of FGF1 for one hour and analyzed for phosphorylated (p) FRS2, ERK and FGFR1 by western blot; total ERK, FGFR1 and tubulin served as loading controls. pFRS2, pERK and pFGFR1 signal was quantified and plotted (mean±SD; t-test, **$p<0.01$, ***$p<0.001$; n, number of independent experiments). (**D**) U2OS cells stably transfected with empty pcDNA3.1 vector (U2OS-pcDNA3.1) and pcDNA3.1 vector encoding human FGF1 C-terminally tagged with Myc (U2OS-FGF1myc) were prepared as described previously[13]. To induce FGF1 secretion, serum-starved cells were incubated at 42°C for 2 hours. The FGF1-conditioned media was collected, centrifuged and concentrated on an Amicon filter (Sigma Aldrich). Conditioned media from U2OS cells transfected

with an empty pcDNA3.1 was used as a control. NIH3T3 cells were incubated with VZ23 for 30 minutes, followed by incubation with media conditioned by U2OS cells for 15 minutes. Cells were lysed with Laemmli sample buffer and analyzed by western blot. The pERK signal was quantified and plotted (mean±SD; t-test, ***p<0.001; n, number of independent experiments). CM, conditioned media.

```
                22   domain Ig1                                         81
hFGFR1          RPSPTLPEQAQPWGAPVEVESFLVHPGDLLQLRCRLRDDVQSINWLRDGVQLAESNRTRI
mFGFR1          RPAPTLPEQAQPWGVPVEVESLLVHPGDLLQLRCRLRDDVQSINWLRDGVQLVESNRTRI
rFGFR1          RPAPTLPEQAQPWGVPVEVESLLVHPGDLLQLRCRLRDDVQSINWLRDGVQLAESNRTRI
                **:***********.******:******************************.*******

                82                                                     141
hFGFR1          TGEEVEVQDSVPADSGLYACVTSSPSGSDTTYFSVNVSDALPSSEDDDDDDDSSSEEKET
mFGFR1          TGEEVEVRDSIPADSGLYACVTSSPSGSDTTYFSVNVSDALPSSEDDDDDDDSSSEEKET
rFGFR1          TGEEVEVRDSIPADSGLYACVTNSPSGSDTTYFSVNVSDALPSSEDDDDDDDSSSEEKET
                *******.**:***********.*************************************

               142                   domain Ig2                        201
hFGFR1          DNTKPNRMPVAPYWTSPEKMEKKLHAVPAAKTVKFKCPSSGTPNPTLRWLKNGKEFKPDH
mFGFR1          DNTKPNRRPVAPYWTSPEKMEKKLHAVPAAKTVKFKCPSSGTPNPTLRWLKNGKEFKPDH
rFGFR1          DNTKPNRRPVAPYWTSPEKMEKKLHAVPAAKTVKFKCPSSGTPSPTLRWLKNGKEFKPDH
                ******* ***********************************.****************

               202                                                     261
hFGFR1          RIGGYKVRYATWSIIMDSVVPSDKGNYTCIVENEYGSINHTYQLDVVERSPHRPILQAGL
mFGFR1          RIGGYKVRYATWSIIMDSVVPSDKGNYTCIVENEYGSINHTYQLDVVERSPHRPILQAGL
rFGFR1          RIGGYKVRYATWSIIMDSVVPSDKGNYTCIVENEYGSINHTYQLDVVERSPHRPILQAGL
                ************************************************************

               262              domain Ig3                             321
hFGFR1          PANKTVALGSNVEFMCKVYSDPQPHIQWLKHIEVNGSKIGPDNLPYVQILKTAGVNTTDK
mFGFR1          PANKTVALGSNVEFMCKVYSDPQPHIQWLKHIEVNGSKIGPDNLPYVQILKTAGVNTTDK
rFGFR1          PANKTVALGSNVEFMCKVYSDPQPHIQWLKHIEVNGSKIGPDNLPYDQILKTAGVNTTDK
                ********************************************** *************

               322                                                376
hFGFR1          EMEVLHLRNVSFEDAGEYTCLAGNSIGLSHHSAWLTVLEALEERPAVMTSPLYLE
mFGFR1          EMEVLHLRNVSFEDAGEYTCLAGNSIGLSHHSAWLTVLEALEERPAVMTSPLYLE
rFGFR1          EMEVLHLRNVSFEDAGEYTCLAGNSIGLSHHSAWLTVLEALEERPAVMTSPLYLE
                *******************************************************
```

Figure S12 Sequence alignment of the extracellular domain of human (h), mouse (m) and rat (r) FGFR1c (UniProt P11362-1, P16092-1, Q04589) with Muscle 3.8. The positions of Ig-like domains 1-3 are indicated; (*) fully conserved, (:) conserved between amino acids classes, (.) weak similarity between amino acid classes, ( ) non-conserved. The alignment demonstrates 97.5%, 98% and 98.9% similarity for hFGFR1:rFGFR1, hFGFR1:mFGFR1 and mFGFR1:rFGFR1 pairs, respectively. Moreover, most of the differences in sequence (6 out of 7 for hFGFR:mFGFR and 6 out of 9 for hFGFR1:rFGFR1) occur in the Ig1 domain, which is not involved in VZ23 binding.

References

1 Guagnano, V.; Furet, P.; Spanka, C.; Bordas, V.; Le Douget, M.; Stamm, C.; Brueggen, J.; Jensen, M. R.; Schnell, C.; Schmid, H.; et al. (2011) Discovery of 3-(2,6-Dichloro-3,5-Dimethoxy-Phenyl)-1-{6-[4-(4-Ethyl-Piperazin-1-Yl)-Phenylamino]-Pyrimidin-4-Yl}-1-Methyl-Urea (NVP-BGJ398), A Potent and Selective Inhibitor of the Fibroblast Growth Factor Receptor Family of Receptor Tyrosine Kinase. *J. Med. Chem. 54* (20), 7066–7083. https://doi.org/10.1021/jm2006222.

2 Liu, P. C. C.; Koblish, H.; Wu, L.; Bowman, K.; Diamond, S.; DiMatteo, D.; Zhang, Y.; Hansbury, M.; Rupar, M.; Wen, X.; et al. (2020) INCB054828 (Pemigatinib), a Potent and Selective Inhibitor of Fibroblast Growth Factor Receptors 1, 2, and 3, Displays Activity against Genetically Defined Tumor Models. *PLoS ONE 15* (4), e0231877. https://doi.org/10.1371/journal.pone.0231877.

3 Hanna, K.S. (2019) Erdafitinib to Treat Urothelial Carcinoma. *Drugs Today (Barc) 55* (8), 495. https://doi.org/10.1358/dot.2019.55.8.3010573.

4 Gavine, P. R.; Mooney, L.; Kilgour, E.; Thomas, A. P.; Al-Kadhimi, K.; Beck, S.; Rooney, C.; Coleman, T.; Baker, D.; Mellor M. J.; et al. (2012) AZD4547: An Orally Bioavailable, Potent, and Selective Inhibitor of the Fibroblast Growth Factor Receptor Tyrosine Kinase Family. *Cancer Res. 72* (8), 2045–2056. https://doi.org/10.1158/0008-5472.CAN-11-3034.

5 Sootome, H.; Fujita, H.; Ito, K.; Ochiiwa, H.; Fujioka, Y.; Ito, K.; Miura, A.; Sagara, T.; Ito, S.; Ohsawa, H.; et al. (2020) Futibatinib Is a Novel Irreversible FGFR 1–4 Inhibitor That Shows Selective Antitumor Activity against FGFR-Deregulated Tumors. *Cancer Res. 80* (22), 4986–4997. https://doi.org/10.1158/0008-5472.CAN-19-2568.

6 Hall, T. G.; Yu, Y.; Eathiraj, S.; Wang, Y.; Savage, R. E.; Lapierre, J.-M.; Schwartz, B.; Abbadessa, G. (2016) Preclinical Activity of ARQ 087, a Novel Inhibitor Targeting FGFR Dysregulation. *PLoS ONE 11* (9), e0162594. https://doi.org/10.1371/journal.pone.0162594.

7 Bello, E.; Colella, G.; Scarlato, V.; Oliva, P.; Berndt, A.; Valbusa, G.; Serra, S. C.; D'Incalci, M.; Cavalletti, E.; Giavazzi, R.; et al. (2011) E-3810 Is a Potent Dual Inhibitor of VEGFR and FGFR That Exerts Antitumor Activity in Multiple Preclinical Models. *Cancer Res. 71* (4), 1396–1405. https://doi.org/10.1158/0008-5472.CAN-10-2700.

8 Watanabe Miyano, S.; Yamamoto, Y.; Kodama, K.; Miyajima, Y.; Mikamoto, M.; Nakagawa, T.; Kuramochi, H.; Funasaka, S.; Nagao, S.; Sugi, N. H.; et al. (2016) E7090, a Novel Selective Inhibitor of Fibroblast Growth Factor Receptors, Displays Potent Antitumor Activity and Prolongs Survival in Preclinical Models. *Mol. Cancer Ther. 15* (11), 2630–2639. https://doi.org/10.1158/1535-7163.MCT-16-0261.

9 Nakanishi, Y.; Akiyama, N.; Tsukaguchi, T.; Fujii, T.; Sakata, K.; Sase, H.; Isobe, T.; Morikami, K.; Shindoh, H.; Mio, T.; et al. (2014) The Fibroblast Growth Factor Receptor Genetic Status as a Potential Predictor of the Sensitivity to CH5183284/Debio 1347, a Novel Selective FGFR Inhibitor. *Mol. Cancer Ther. 13* (11), 2547–2558. https://doi.org/10.1158/1535-7163.MCT-14-0248.

10 Babina, I. S.; Turner, N. C. (2017) Advances and Challenges in Targeting FGFR Signalling in Cancer. *Nat. Rev. Cancer 17* (5), 318–332. https://doi.org/10.1038/nrc.2017.8.

11 Subbiah, V.; Sahai, V.; Maglic, D.; Bruderek, K.; Touré, B. B.; Zhao, S.; Valverde, R.; O'Hearn, P. J.; Moustakas, D. T.; Schönherr, H.; et al. (2023) B. RLY-4008, the First Highly Selective FGFR2 Inhibitor with Activity across *FGFR2* Alterations and Resistance Mutations. *Cancer Discov. 13* (9), 2012–2031. https://doi.org/10.1158/2159-8290.CD-23-0475.

12 Zhou, Z.; Chen, X.; Fu, Y.; Zhang, Y.; Dai, S.; Li, J.; Chen, L.; Xu, G.; Chen, Z.; Chen, Y. (2019) Characterization of FGF401 as a Reversible Covalent Inhibitor of Fibroblast Growth Factor Receptor 4. *Chem. Commun.* **2019**, *55* (42), 5890–5893. https://doi.org/10.1039/C9CC02052G

13 Biadun, M., Sochacka, M., Kalka, M., Chorazewska, A., Karelus, R., Krowarsch, D., Opalinski, L., and Zakrzewska, M. (2024). Uncovering key steps in FGF12 cellular release reveals a common mechanism for unconventional FGF protein secretion. Cell. Mol. Life Sci. *81*, 356. https://doi.org/10.1007/s00018-024-05396-9.
